# Relative Abundance of Integral Plasma Membrane Proteins in Arabidopsis Leaf and Root Tissue Determined by Metabolic Labeling and Mass Spectrometry

**DOI:** 10.1371/journal.pone.0071206

**Published:** 2013-08-19

**Authors:** Katja Bernfur, Olaf Larsson, Christer Larsson, Niklas Gustavsson

**Affiliations:** 1 Department of Biochemistry and Structural Biology, Center for Molecular Protein Science, Lund University, Lund, Sweden; 2 Mutation Analysis Facility, Clinical Research Centre, Novum, Huddinge University Hospital, Stockholm, Sweden; 3 Novo Nordisk A/S, Novo Nordisk Park, Måløv, Denmark; University of Technology Sydney, Australia

## Abstract

Metabolic labeling of proteins with a stable isotope (^15^N) in intact Arabidopsis plants was used for accurate determination by mass spectrometry of differences in protein abundance between plasma membranes isolated from leaves and roots. In total, 703 proteins were identified, of which 188 were predicted to be integral membrane proteins. Major classes were transporters, receptors, proteins involved in membrane trafficking and cell wall-related proteins. Forty-one of the integral proteins, including nine of the 13 isoforms of the PIP (plasma membrane intrinsic protein) aquaporin subfamily, could be identified by peptides unique to these proteins, which made it possible to determine their relative abundance in leaf and root tissue. In addition, peptides shared between isoforms gave information on the proportions of these isoforms. A comparison between our data for protein levels and corresponding data for mRNA levels in the widely used database Genevestigator showed an agreement for only about two thirds of the proteins. By contrast, localization data available in the literature for 21 of the 41 proteins show a much better agreement with our data, in particular data based on immunostaining of proteins and GUS-staining of promoter activity. Thus, although mRNA levels may provide a useful approximation for protein levels, detection and quantification of isoform-specific peptides by proteomics should generate the most reliable data for the proteome.

## Introduction

The plasma membrane constitutes the outer border of the cell. This position implies that properties of the plasma membrane will determine almost all types of communication and other interactions between the cell interior and its surrounding environment. Communication is controlled by proteins either embedded in the plasma membrane bilayer (integral proteins) or associated with its inner or outer surface (peripheral proteins), such as transport proteins, proteins involved in membrane trafficking and signal transduction components. Due to the very diverse functions of different organs and tissues, the plasma membrane is likely to be the most diverse membrane of the cell, with a protein composition that varies according to its location in the plant, as well as to developmental stage and environment. We have chosen to focus on integral membrane proteins, as predicted by Phobius [1], and compared the protein composition of plasma membranes isolated from leaf and root tissue from eight-week-old Arabidopsis plants grown on a liquid medium. At this age, the leaf rosette contains leaves of all developmental stages, from fully expanded to newly developed, and the root system is still growing. The relative abundance of proteins in leaves and roots was determined by metabolic labeling of proteins with a stable isotope (^15^N) and subsequent proteomic analysis using mass spectrometry (MS).

Labeling of proteins with stable isotopes has emerged within proteomic research as an accurate method for determination of differences in protein abundance among samples. Metabolic labeling is often preferred since the incorporation of the stable isotope (e.g., ^13^C or ^15^N) is performed *in vivo* by the organism itself and does not involve any steps that may introduce errors due to incomplete enzymatic or chemical reactions [2].

Below, we have used metabolic labeling of proteins with a stable isotope (^15^N) in intact Arabidopsis plants and subsequent determination of the relative abundance of proteins using MS. To facilitate processing of the large datasets of MS signals, we developed software in house, which automatically calculates the relative abundance of ^14^N- and ^15^N-labeled peptides. This software is available by email (katja.bernfur@biochemistry.lu.se) and at our website where also all primary MS data concerning this project are deposited: http://www.cmps.lu.se/biostruct/people/katja_bernfur/plasma_membrane_proteomics/

## Materials and Methods

### Plant growth

Wild type *Arabidopsis thaliana* ecotype Columbia (Col-0) were grown hydroponically. Seeds were surface sterilized in 95% ethanol followed by 50% bleach+0.05% Tween before germinated and grown on 9-cm plates with Murashige and Skoog [3] mineral salts medium (0.5× MS; pH 5.7) supplemented with 0.8% Bacto-agar for 4 weeks. In the ^15^N enriched medium the normal nitrogen sources were exchanged for 98% K^15^NO_3_ and ^15^NH_4_NO_3_ (Sigma 335134 and 299278, respectively). After 4 weeks the seedlings were transferred from the gel plates to round plastic discs (ø 6 cm) with two holes in the middle where two plants were placed. The discs were placed on top of plastic petri dishes (ø 5.5 cm) containing 30 ml medium keeping the leaves on top of the disc and the roots beneath the medium surface. ^14^N- and ^15^N-plants were grown next to each other in a small greenhouse placed in a room at 22±2°C for a 14/10 h day/night period. When plants were 8 weeks old they were harvested at nine o'clock in the morning (two hours into the light period) and divided into green leaf rosette and root tissue samples. Preparations of leaf and root plasma membranes from ^14^N- and ^15^N-plants were then run in parallel as described below.

### Plasma membrane isolation

All steps in the preparation procedure were performed at 4°C or on ice. Ten g of fresh plant material, either leaf or root tissue, was homogenized with a knife blender in 25 ml homogenization buffer: 0.33 M sucrose, 50 mM MOPS-KOH, pH 7.5, 5 mM EDTA, 0.2% (w/v) casein hydrolysate, 250 µl protease inhibitor cocktail (SIGMA P 9599), including 0.6% (w/v) polyvinylpolypyrrolidone, 5 mM ascorbate and 5 mM dithiothreitol (DTT) added immediately before use. The homogenate was filtered through a nylon mesh (200 µm) and phenylmethanesulphonylfluoride was added to a final concentration of 0.5 mM. The filtrate was centrifuged at 10,000 *g* for 15 min, the pellet was discarded and the supernatant was centrifuged at 50,000 *g* for 30 min. The supernatant, containing soluble proteins, was saved and the microsomal pellet was resuspended in 2 ml resuspension buffer: 0.33 M sucrose, 5 mM K-phosphate, pH 7.8, 0.1 mM EDTA, and 1 mM DTT freshly added. To produce an aqueous polymer two-phase system with a final weight of 8.00 g, the resuspended pellet (2.00 ml) was added to a 6.00 g phase mixture producing a 6.1% (w/w) dextran 500, 6.1% (w/w) polyethylene glycol 3350, 0.33 M sucrose, 5 mM K-phosphate, pH 7.8, 3 mM KCl phase system. Further purification of the plasma membranes using the aqueous polymer two-phase system was performed according to Larsson et al. [4]. The final upper phase, highly enriched in plasma membranes, was diluted two-fold with 0.33 M sucrose, 5 mM K-phosphate, pH 7.8, 0.1 mM EDTA before centrifugation at 100,000 *g* for 1 h. The plasma membrane pellet was resuspended in 200 µl resuspension buffer. Protein concentration was determined according to Bradford [5] with bovine serum albumin (BSA) as a standard.

### SDS-PAGE and immunoblotting

**Figure 1:**
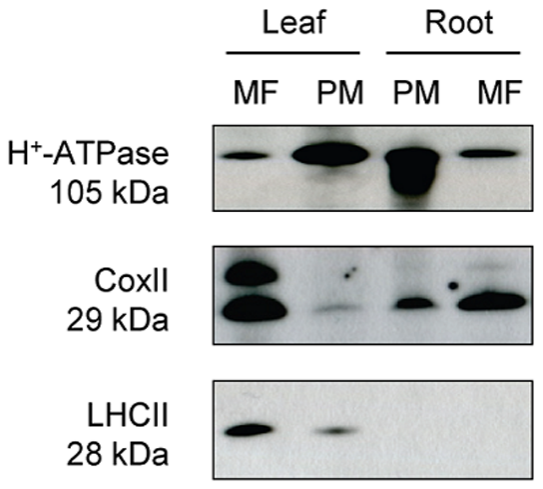
Immunostaining of marker proteins in Arabidopsis membrane fractions. Arabidopsis microsomal fractions (MF) obtained from leaves and roots, respectively, were subjected to aqueous two-phase partitioning to produce plasma membrane (PM) fractions. Polypeptides were separated by SDS-PAGE (7 µg of protein per lane), transferred to a blotting membrane, and immunostained with antisera directed against the plasma membrane H^+^-ATPase, subunit II of the mitochondrial cytochrome oxidase (CoxII), and light harvesting complex II (LHCII) of the chloroplast thylakoid membrane. Molecular weights (kDa) are indicated to the left. Note that the antiserum against CoxII also produced a band at a slightly higher molecular weight than the expected 29 kDa.

Samples were solubilized at room temperature in standard sample buffer, and polypeptides were separated by SDS-PAGE (12% acrylamide, 0.3% bisacrylamide) according to Laemmli [6]. Gels were either stained with colloidal Coomassie [7], or polypeptides were electrophoretically transferred to an Immobilon polyvinylidene difluoride transfer membrane (Millipore, USA) for immunostaining. After blocking in 2% (w/v) BSA in phosphate buffered saline (PBS: 0.15 M NaCl, 0.01 M potassium phosphate, pH 7.5) overnight, the blots were incubated with one of the following rabbit polyclonal antisera diluted in PBS: 1) Anti-Lhcb1 (Agrisera, Vännäs, Sweden), an antiserum raised against a peptide corresponding to a sequence of the Lhcb1 protein (one of three LHCII isoforms) of Arabidopsis. 2) Anti-COXII (Agrisera, Vännäs, Sweden), an antiserum raised against a peptide corresponding to a widely conserved sequence of the mitochondrial cytochrome oxidase subunit II. 3) Anti-H^+^-ATPase, an antiserum raised against a polypeptide corresponding to amino acids 851 to 949 of the C terminus of the Arabidopsis H^+^-ATPase isoform 2 (AHA2) and a kind gift from Professor R. Serrano (Universidad Politecnica, Valencia, Spain) (Figure 1). The horseradish peroxidase-conjugated secondary antibody was visualized by enhanced chemiluminescence (GE Healthcare, Buckinghamshire, UK).

### In-gel digestion

Each lane on the SDS-gel was divided into 36 segments for trypsin digestion. The gel segments were placed in 96-well plates and washed twice in 75 µl 50 mM ammonium bicarbonate, 50% ethanol buffer to remove the commassie staining. After dehydration in 75 µl 100% ethanol, the gel pieces were subjected to reduction and alkylation by adding 10 µl of 10 mM DTT and 10 µl of 55 mM iodoacetamide with a second dehydration step in between. The gel pieces were washed and dehydrated once again before 8 µl of digestion buffer (10 ng/µl trypsin proteomics grade in 50 mM NH_4_HCO_3_) was added. Finally, 15 µl of 50 mM NH_4_HCO_3_ was added and the samples were digested overnight at 37°C. Peptides were extracted by adding 15 µl of 1% trifluoroacetic acid (TFA) and incubation for 2 h. The supernatant was collected and pooled with the overnight digestion solution.

### Liquid chromatography

Peptides from the in-situ digestion were separated by reversed phase nano-LC using a 1100 Series Nanoflow LC system (Agilent Technologies, Waldbronn, Germany) with mobile phase buffers A [1% (v/v) acetonitrile and 0.1% TFA] and B (90% acetonitrile and 0.1% TFA). Eight µl of the peptide extract was loaded on to a precolumn (Zorbax 300 SB C_18_, 5×0.3 mm) and then separated on a separation column (Zorbax 300 SB C_18_, 150×0.075 mm) at a flow rate of 0.3 µl/min. Fractions were collected on a stainless steel MALDI-target using the 1100 Series LC micro collection spotting system. When collecting 48 fractions, peptides are eluted with the following elution profile: 0 to 10 min, 0% B: 10 to 17 min, 10% B: 17 to 70 min, 10 to 70% B: 70 to 76 min, 100% B: 76 to 82 min, 0% B. Fraction collection was performed from 28 min to 61 min in 0.7 min intervals. Mixed ^14^N and ^15^N fractions contain almost double the amount of peptides. To reduce sample complexity these samples were collected in 192 fractions with the following elution profile: 0 to 9 min, 0% B: 9 to 17 min, 10% B: 17 to 84 min, 10 to 80% B: 84 to 86 min, 80 to 100% B: 86 to 96 min, 100% B: 96 to 106 min, 0% B. Fraction collection was performed from 28 min to 56,5 min in 0,15 min intervals.

### Mass spectrometry, MS/MS and protein identification

MS and MS/MS spectra were acquired on a 4700 MALDI TOF/TOF mass spectrometer (Applied Biosystems, Framingham, CA, USA) in positive reflector mode. Matrix solution, 0.5 µl containing 5 mg/ml α-cyano-4-hydroxy cinnamic acid, 50% acetonitrile, 1% TFA and 25 mM citric acid, was added onto the dried peptide sample eluted from the LC. For identification of plasma membrane proteins in leaf and root tissues, ^14^N samples were LC-fractionated on 48 spots and MS spectra were recorded automatically before MS/MS spectra were acquired for the ten most intense ions in each MS spectrum. The jobwide interpretation function in the Peak Explorer software was used to select the precursor ions where the minimum signal to noise value for selection was set to 60 and the spot-to-spot difference between precursor masses to +/−100 ppm. Known trypsin autodigestion peptides and keratin peptides were excluded. Mixed ^15^N and ^14^N samples, LC fractionated on 192 spots, were recorded automatically in MS mode. Totally 3000 to 4000 shots were collected per spectrum to achieve good ion statistics. All MS spectra were internally calibrated using three standard peptides, Angiotensin II, m/z 1046.541; Neurotensin, m/z 1672.91 and ACTH 18–39, m/z 2465.199. GPS Explorer software, an in-house Mascot search engine (Matrix Science, London, UK) and the TAIR protein database were used for protein identifications in the leaf and root ^14^N fractions using the acquired MS/MS data. The settings used for the database search were: trypsin-specific digestion, digestion with one missed cleavage site, peptide mass tolerance set to +/−30 ppm, fragment ion mass tolerance set to +/−0.3 Da, carbamidomethylation set as fixed modification. To regard the protein as identified, a confidence level of 99.5% on the protein level was used.

### Data processing

**Figure 2:**
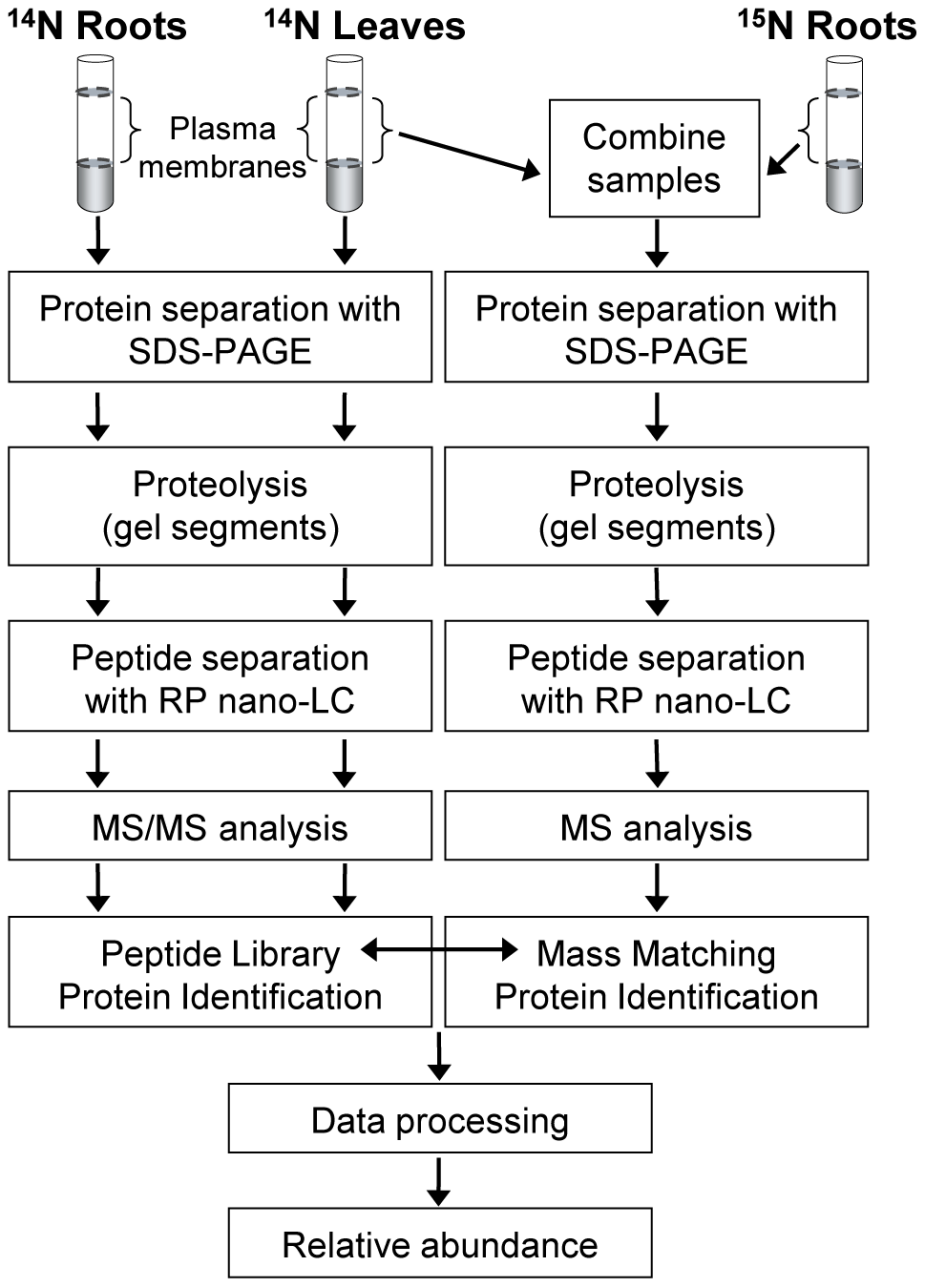
Workflow. Plasma membrane fractions (or, in one case, soluble protein fractions, Fig. 4) were prepared from leaf and root tissue, respectively, obtained from intact Arabidopsis plants hydroponically grown either on a ^14^N or a ^15^N medium. Left panel: Proteins were separated by SDS-PAGE and protein bands were excised for proteolysis. Peptides were separated by reversed-phase nano-liquid chromatography (RP nano-LC) and analyzed by MALDI-TOF MS/MS for peptide and protein identification, and construction of a peptide library. Right panel: leaf and root plasma membrane preparations were combined at a 1∶1 protein ratio and proteins were separated by SDS-PAGE. Protein bands were excised, digested, and peptides were separated by RP nano-LC and analyzed by MALDI MS. Finally, the relative abundance of individual proteins in leaf and root plasma membranes was determined from the signal intensities of the ^14^N/^15^N peptide pairs (Compare Fig. 4, below).

Our in-house developed software allows for automated processing of the large MS data sets obtained after metabolic labeling of proteins. The software can for instance be used to determine the relative abundance of proteins in different samples labeled with ^14^N/^15^N isotopes. In the present work, proteins were first identified from leaf and root samples with one single (^14^N) isotopic distribution by searching the TAIR protein database. Then, peptide information from the identified proteins was used for scanning through MS data from samples containing proteins with different (^14^N/^15^N) isotopic distributions (Workflow, Figure 2).

**Figure 3:**
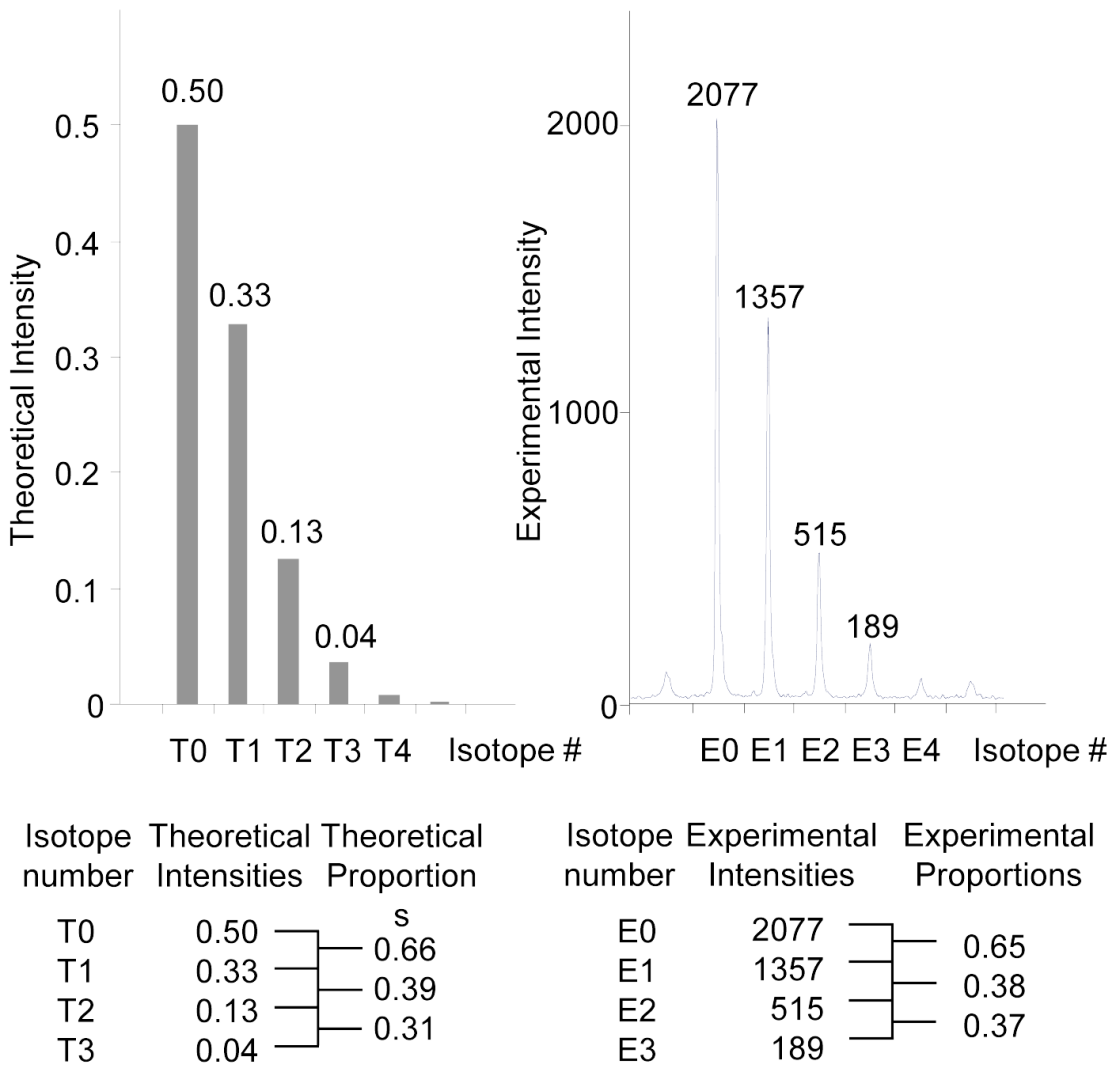
Consistency between theoretical and experimental isotope signal envelopes. Comparisons of intensity ratios for neighboring isotope signals in an experimentally obtained envelope with the corresponding intensity ratios for a theoretical envelope assuming 98.2% ^15^N labeling efficiency. Left panel: theoretical isotope pattern for the peptide DVEGPEGFQTR from the aquaporin PIP2;2. Right panel: experimental isotope pattern for the same peptide. The difference from the theoretical ratio was calculated for each pair of signals and only isotopic envelopes that did not differ more than the threshold values preset in the settings file of the processing software were used for further analysis (calculations below the panel).

Briefly, the program reads a file with information about each identified protein and peptide derived from them, and peak list files with MS data, which contain the signal intensity and corresponding mass for each detected signal. Various parameters for the program are specified in a settings panel which allows to adjust mass tolerance, isotopic pattern correlation, etc. The program reads the peptide sequences for each protein identified in the TAIR database search and calculates the theoretical masses for the peptides, based on the atomic composition of the peptides amino acids. The program also generates theoretical isotope distributions for each peptide, based on the peptide atomic composition, and data regarding the natural occurrence of isotopes. It uses this information to compare the theoretical isotopic distributions with the experimental ones, to get a quality measurement of the experimental data. Theoretical isotope distributions are calculated for naturally occurring ^14^N peptides as well as ^15^N-labelled peptides (Figure 3). Based on the information about the theoretical masses of the peptides, the program then searches all the peak lists for signals from these peptide masses and extracts signal data in the relevant m/z-window for further processing.

**Figure 4:**
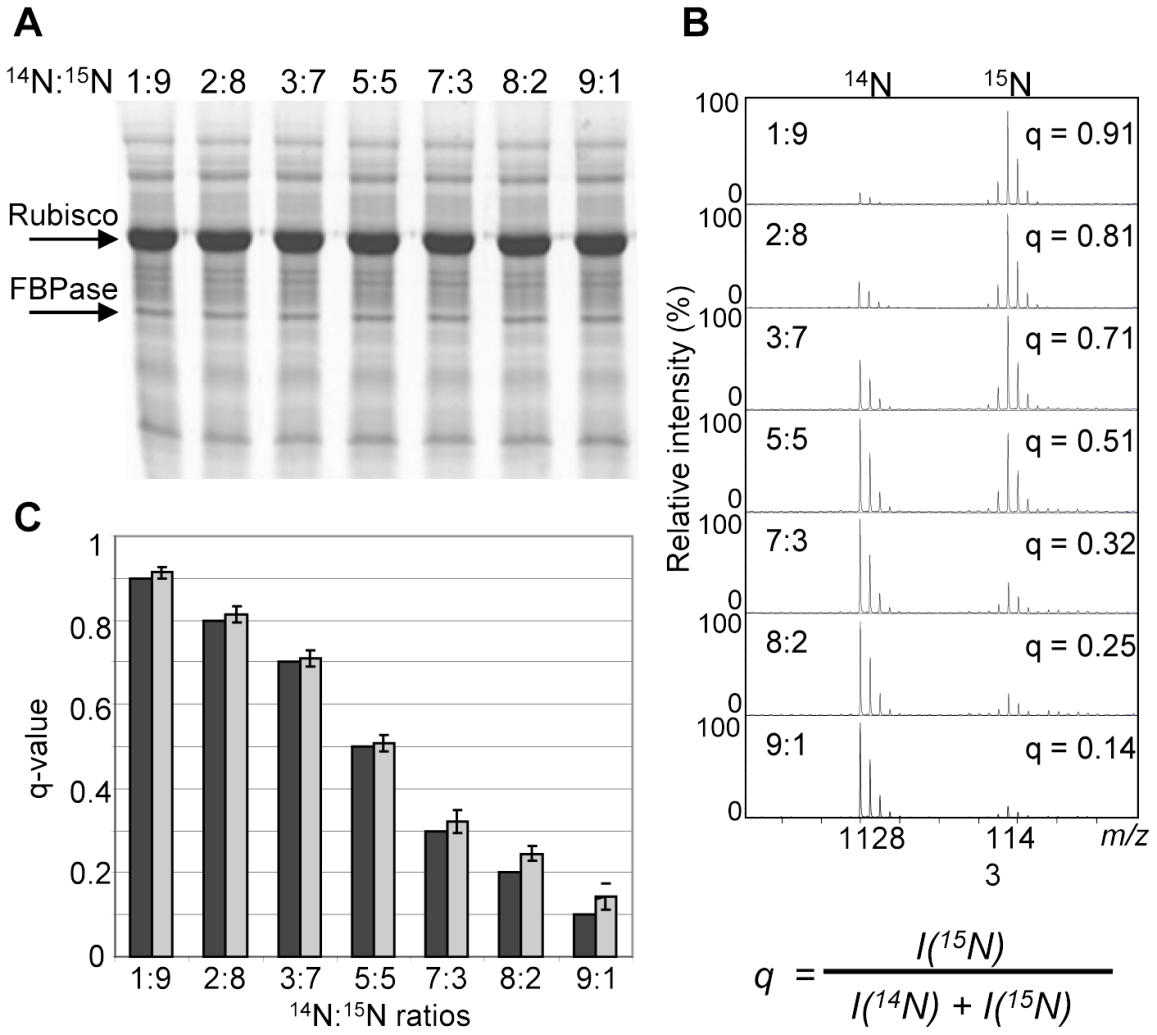
Determination of the range within which reliable quantitative data can be obtained. A, Soluble leaf protein extracts from plants grown in ^14^N - and ^15^N -medium, respectively, were mixed at different ratios (1∶9, 2∶8, 3∶7, 5∶5, 7∶3, 8∶2, 9∶1) and subjected to SDS-PAGE. Two bands containing Rubisco large subunit, and fructose-1,6-bisphosphate aldolase (FBPase) were excised from each lane and processed as described in Figure 2. B, Experimental data for one of the FBPase peptides (TAAYYQQGAR). C, Average q-values for 11 peptides (6 from Rubisco and 5 from FBPase, in total based on 94 spectra) are summarized (grey columns; bar = standard deviation) and compared to the theoretical values (black columns). The relative abundance of ^14^N - and ^15^N -labeled peptides is defined as the fraction of ^15^N -labeled species of total peptide = the q-value of the peptide (equation at bottom, right. I = Intensity).

Results are printed to a main output folder, in which subfolders for each protein are generated. The subfolders are labelled with the accession number of the protein, for which information they contain. Each of the protein subfolders contains result files for all of the peptides that have been matched to this protein. For each peptide the program produces a result file containing quantitative information about each peptide pair found in the data set. For each peptide pair, signals in the relevant m/z-window are extracted from all peak lists and are shown in the results file for this peptide. The result is presented as the sum of the signals from the heavy isotope peptide divided by the sum of all signals of the light and heavy peptide (q-value; Figure 4). Each protein folder containing data on peptide results also includes a q-value plot. For each protein, good quality q-values for every peptide pair from all peak lists are shown as a dot in the q-value plot, which makes it possible to get an overview of the q-value results for this protein.

## Results and Discussion

### The plasma membrane preparations

Plasma membranes were prepared by aqueous polymer two-phase partitioning [8] using a phase system where plasma membranes partition to the upper phase and intracellular membranes partition to the interface and to the lower phase [4]. Plasma membranes prepared by this technique are usually of high purity (about 95%) and consist mainly of right-side-out (cytoplasmic-side-in) vesicles [4]. The yield of plasma membrane protein was approximately 0.25 and 0.05 mg from leaves and roots, respectively, corresponding to 5 and 3% of total microsomal membrane protein, respectively. To obtain a measure of the degree of purification of the plasma membrane fractions, immunostaining of the H^+^-ATPase, a canonical plasma membrane protein [9], was compared between the plasma membrane and microsomal fractions (Figure 1). A strong enrichment of the H^+^-ATPase was observed for both plasma membrane preparations. Leaf microsomal fractions are usually dominated by chloroplast membranes, whereas root microsomal fractions are usually dominated by mitochondrial membranes [10]. Therefore, immunostaining of light harvesting complex II (LHCII), a major component of the chloroplast thylakoid membrane, and cytochrome oxidase (subunit II), a major component of the mitochondrial inner membrane, was used to determine the degree of contamination by these membranes.

As seen in Figure 1, some contamination with mitochondrial membranes was found, particularly in the root plasma membranes, whereas some contamination with thylakoid membranes was found in the leaf plasma membranes. However, no integral proteins belonging to the inner mitochondrial membrane or to the chloroplast thylakoid membrane were identified by MS. Although present, as shown by the immunoblots in Figure 1, these proteins obviously fell below the detection limit in the mass spectrometer. A low degree of contamination was suggested also by the MS data and no marker proteins for any other membrane than the plasma membrane were detected (Table S1).

### Development of the method used

The workflow used is outlined in Figure 2. In the workflow, plasma membranes from leaves and roots obtained from plants grown on ^14^N-medium were used for the initial identification of proteins and the construction of a corresponding peptide library. The identification of these peptides was based on MS/MS data used for database searches with high stringency, total ion score >99.5% (left panel in Figure 2). Amino acid sequences from the peptide identification results were used to calculate the theoretical ^14^N as well as ^15^N masses of each peptide. These theoretical masses were then used to find ^14^N/^15^N peptide pairs in the mixed sample spectra (right panel in Figure 2). However, as matching of masses between the peptide library and peptides in the combined samples was based on MS data only (right panel in Figure 2), many false positive matches were detected, due to the fact that one mass can fit many different amino acid sequences with the given mass accuracy of the MS instrumentation used. When a protein is present in both tissues this problem is less pronounced since both ^14^N- and ^15^N-labeled peptide masses need to agree with the specific number of nitrogen present in the peptide sequence. The risk of getting a false positive hit is therefore higher when a protein is present in only one tissue and therefore only generates one, either ^14^N- or ^15^N-, peptide signal. Such false positives were identified and removed from the data set by introducing two additional criteria. To be regarded as a true match, the peptide identified by MS only should 1) originate from the same segment of the SDS-polyacrylamide gel as the MS/MS identification had been done, and from a position where the full size protein would band theoretically, and 2) elute at a similar position in the gradient used for nano-LC separation as the peptide identified by MS/MS. Furthermore, all spectra that fulfilled all criteria were manually inspected to confirm that the isotopic pattern was correct.

To establish the workflow described above and to evaluate the data and its accuracy, a number of initial experiments were performed to determine ^15^N-labeling efficiency etc. Labeling efficiency was determined by comparing the isotopic signal envelope for the ^15^N-enriched peptides with theoretical isotopic signal envelopes calculated for different degrees of ^15^N incorporation from 90 to 100%. ^15^N incorporation was found to be 98.2% for proteins in both leaf and root tissue and this value was used in the settings file for the software used for the calculations of relative protein abundance. Besides matching experimental masses of peptides in the peak lists from the combined ^14^N/^15^N samples to the theoretical masses calculated from the amino acid sequences of peptides used for protein identification, we also developed a numerical method for matching the experimental isotopic signal envelope to the theoretical isotopic signal envelope obtained at 98.2% ^15^N labeling efficiency. This was done by pairwise comparisons of intensity ratios for neighboring isotope signals in an experimentally obtained envelope with the corresponding intensity ratios for a theoretical envelope. In Figure 3, the left panel shows the theoretical isotope pattern for the peptide DVEGPEGFQTR from the aquaporin PIP2;2 and the right panel shows the experimental isotope pattern of the same peptide. The difference from the theoretical ratio was calculated for each pair of signals (calculations below the panel) and only isotopic envelopes that did not differ more than the threshold values preset in the settings file of the processing software were used for further analysis. This was used to eliminate spectra containing overlapping peptide pair signals as well as spectra for peptides matching theoretical masses but with a different amino acid composition.

To determine the range within which reliable quantitative data can be obtained, as well as the precision of the software, a series of samples were prepared by mixing soluble leaf protein extracts from plants grown in ^14^N- and ^15^N-medium, respectively, at different ratios (1∶9, 2∶8, 3∶7, 5∶5, 7∶3, 8∶2, 9∶1). The mixed samples were separated by SDS-PAGE and two bands (Figure 4) were selected, excised from each lane and processed according to the workflow in Figure 2. Among the proteins identified in the two bands were the large subunit of Ribulose-1,5-bisphosphate carboxylase/oxygenase (Rubisco; ATCG00490) and fructose-1,6-bisphosphate aldolase (FBPase; AT4G38970). The q-values (definition: Figure 4, bottom right) for six peptide pairs matching Rubisco and five peptide pairs matching FBPase were determined from the data obtained from each lane. An example of one such peptide pair from FBPase for which quantitative data was obtained is shown in Figure 4B with the experimentally determined q-values indicated in each panel. The agreement between the experimentally determined q-values and the expected theoretical values for all 11 peptide pairs (based on 94 spectra) is shown in Figure 4C. Note that we have defined the relative abundance of ^14^N- and ^15^N-labeled peptides as the fraction of ^15^N-labeled species (equation in Figure 4) and refer to this as the q-value for that peptide. Thus, a q-value of 0.5 indicates that the protein is present at equal abundance in the two samples. A q-value of 0.6 indicates that the protein is 50% more abundant in the ^15^N-labeled sample compared to the ^14^N-labeled sample, and a q-value of 0.8 means that the protein is 4 times as abundant in the ^15^N-labeled sample compared to the ^14^N-labeled sample. For less abundant peptides, q-values>0.8 and <0.2 are considered to be uncertain, due to the low signal strength of either the ^14^N- or ^15^N-labeled peptide (Compare Figure 4B) and may be interpreted as 1 and 0, respectively.

**Figure 5:**
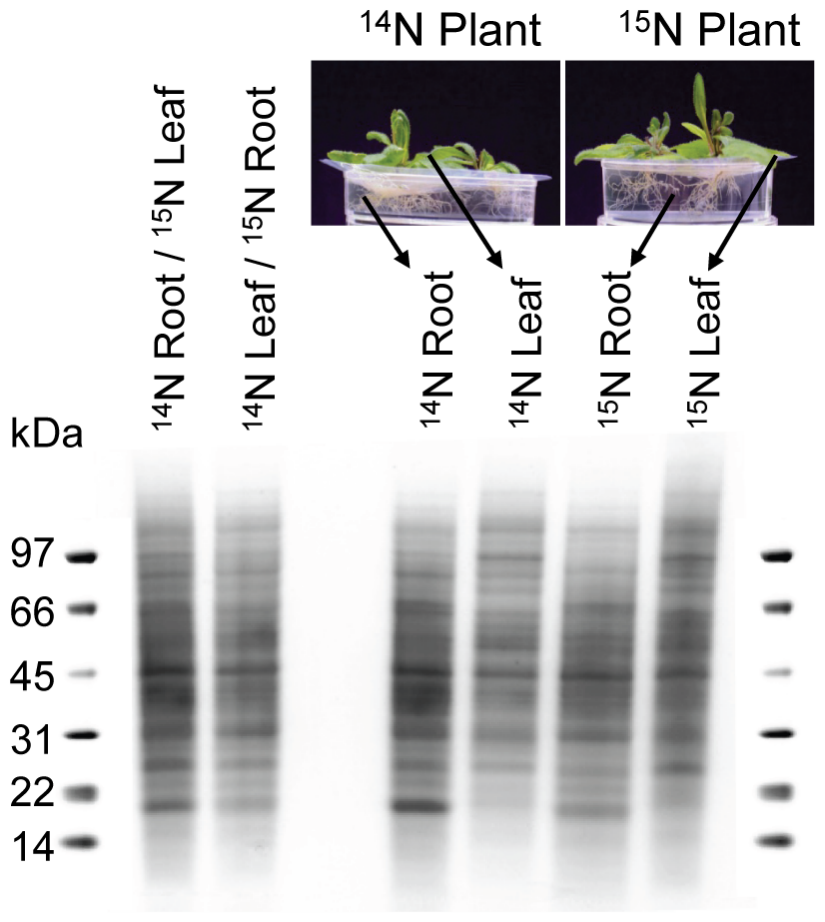
SDS-PAGE of Arabidopsis plasma membranes. Arabidopsis plants were grown on ^14^N- and ^15^N-media, respectively. Leaf and root plasma membranes were isolated and subjected to SDS-PAGE (right), as well as 1/1 mixtures of ^14^N-leaf and ^15^N-root plasma membranes and vice versa (left).

There were no differences between plants grown on ^14^N- and ^15^N-media visible to the naked eye, and SDS-PAGE showed very similar polypeptide patterns for leaf and root plasma membranes, respectively, irrespective of growth medium (Figure 5, right). Such a similarity in polypeptide patterns was first reported for plasma membranes derived from leaves and roots of barley [10] and has later also been observed for plasma membranes from other tissues, such as leaves, xylem, and cambium/phloem from poplar trees [11], suggesting that there is a basic set of proteins common to all plasma membranes, which was confirmed by proteomics in the poplar study [11].

**Figure 6:**
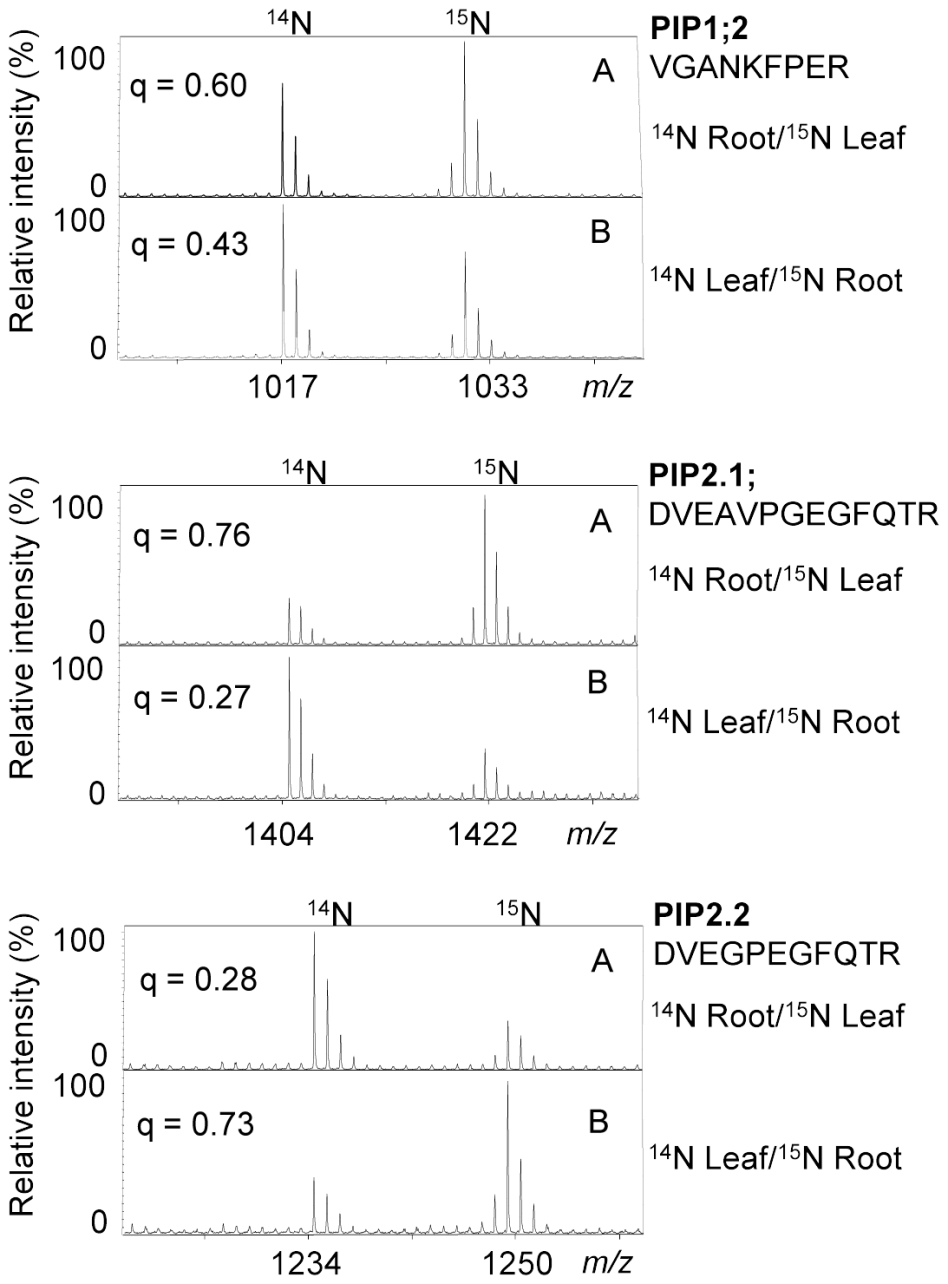
Spectra and q-values for ^14^N/^15^N-peptide pairs. Spectra and comparison of q-values for three ^14^N/^15^N-peptide pairs matching three PIP isoforms. The peptide pairs were from experiments in which either the leaf material was from ^14^N plants and the root material was from ^15^N plants, or vice versa (compare Figure 5).

To exclude differences at the protein level caused by growth on the two different media, plasma membranes from leaves obtained from plants grown in ^14^N and from roots obtained from ^15^N plants, and vice versa, were mixed at a ratio of 1∶1 and subjected to SDS-PAGE (Figure 5, left). Different isoforms of the aquaporin protein family were identified in both leaf and root tissue, mainly members of the plasma membrane intrinsic protein (PIP) subfamily. In Figure 6 we show spectra for three ^14^N/^15^N-peptide pairs matching three different PIP isoforms from experiments in which either the leaf material was from ^14^N plants and the root material was from ^15^N plants, or vice versa. The obtained q-values for each ^14^N/^15^N- and ^15^N/^14^N set of peptide pairs correlate very well with each other, which indicates that growth on ^15^N medium did not affect the protein composition of the plants compared to growth on ^14^N medium.

### Relative abundance of integral membrane proteins

We were able to identify 188 integral proteins (Table S1). Major classes were transporters (45), receptors (48), proteins involved in membrane trafficking (18) and cell wall-related proteins (11), in good agreement with previous proteomic studies on plant plasma membranes [11], [12], [13], [14], [15], [16], [17]. However, only 41 of these 188 integral proteins were identified by unique peptides which also generated reliable ^14^N/^15^N spectra. Only peptides unique to a protein can give information on the distribution of that protein, whereas peptides shared between two or more members of a protein family may give additional information. This is well illustrated by our data for the members of the PIP subfamily, belonging to the major intrinsic protein (MIP) superfamily (Table 1).

**Table 1:** Unique and shared peptides from Arabidopsis PIP (plasma membrane Intrinsic protein) aquaporin isoforms and their relative abundance in leaf and root plasma membranes (q-value).

Peptide^a^	AGI Code	Isoform	Q-value (SD)^b^	No. of Spec^c^	GV^d^
QYQALGGGANTVAHGYTK	AT3G61430	PIP1;1	0.60 (0.02)	12	lR
QYQALGGGANTIAHGYTK	AT2G45960	PIP1;2	0.34 (0.03)	23	LR
QPIGTAAQTESK	AT4G23400	PIP1;5	1.00 (0.00)	2	LR
AKDVEAVPGEGFQTR	AT3G53420	PIP2;1	0.30 (0.04)	104	Lr
DVEAVPGEGFQTR	AT3G53420	PIP2;1	0.29 (0.02)	47	Lr
AFQSSYYDR	AT2G37170	PIP2;2	0.74 (0.02)	9	lR
AKDVEGPEGFQTR	AT2G37170	PIP2;2	0.73 (0.02)	48	lR
DVEGPEGFQTR	AT2G37170	PIP2;2	0.71 (0.03)	39	lR
AKDVEGPDGFQTR	AT2G37180	PIP2;3	1.00 (0.00)	2	lR
ALGSFGSFGSFR	AT5G60660	PIP2;4	1.00 (0.00)	4	R
DLDVNESGPPAAR	AT5G60660	PIP2;4	1.00 (0.00)	55	R
SFGAAVIYNNQK	AT2G39010	PIP2;6	0.00 (0.00)	6	L
VFQSTYYNR	AT2G39010	PIP2;6	0.00 (0.00)	7	L
TPYNTLGGGANTVADGYSK	AT4G35100	PIP2;7	0.37 (0.04)	15	LR
QPIGTSAQSDKDYK	AT3G61430, AT2G45960	PIP1;1. PIP1;2	0.39 (0.03)	12	
SFGAAVIYNK	AT3G53420, AT2G37170	PIP2;1. PIP2;2	0.46 (0.05)	26	
AFQSSYYTR	AT3G53420, AT5G60660	PIP2;1. PIP2;4	0.37 (0.05)	39	
WSLYR	AT2G37170, AT2G37180	PIP2;2. PIP2;3	0.73 (0.00)	1	
ALGSFR	AT3G54820, AT4G35100	PIP2;5. PIP2;7	0.41 (0.01)	3	
SFGAAVIYNNEK	AT5G60660, AT4G35100, AT2G16850	PIP2;4. PIP2;7. PIP2;8	0.37 (0.06)	7	
SLGSFR	AT4G23400, AT2G37170, AT2G37180	PIP2;1. PIP2;2. PIP2;3.	0.46 (0.04)	44	
SWSFYR	AT1G01620, AT4G00430, AT4G23400, AT4G35100	PIP1;3. PIP1;4. PIP1;5. PIP2;7	0.44 (0.02)	15	
VGANKFPER	AT3G61430, AT2G45960, AT1G01620, AT4G00430, AT4G23400	PIP1;1. PIP1;2. PIP1;3. PIP1;4. PIP1;5	0.41 (0.02)	111	
SLGAAIIYNK	AT3G61430, AT1G01620, AT4G00430, AT4G23400, AT3G54820	PIP1;1. PIP1;3. PIP1;4. PIP1;5. PIP2;5	0.51 (0.02)	8	
WSFYR	AT1G01620, AT4G00430, AT4G23400, AT3G53420, AT3G54820, AT2G39010, AT4G35100, AT2G16850	PIP1;3. PIP1;4. PIP1;5. PIP2;1. PIP2;5. PIP2;6. PIP2;7. PIP2;8	0.26 (0.02)	9	

a Peptide sequences for which reliable ^14^N/^15^N spectra could be obtained.

b The q-value is a measure of the distribution of the peptide between root and leaf plasma membranes: A q-value of 1 means that the peptide is found in roots only; 0, in leaves only. SD is the standard deviation for the q-value of the peptide based on all spectra containing the ^14^N/^15^N peptide pair for that specific peptide.

c Number of MS spectra containing reliable data for the ^14^N/^15^N peptide pair used to determine the q-value. All these spectra have fulfilled all criteria for that specific peptide/protein, including correct position of the protein on the SDS-gel, consistent elution of the peptide in the nano-LC gradient, and correct peptide molecular mass as well as isotopic pattern.

d Genevestigator data for mRNA distribution between Arabidopsis leaf rosettes and roots converted to a simple letter code: L, mRNA found in leaves only, R, in roots only; LR, about equally distributed between leaves and roots; Lr, mainly in leaves; lR, mainly in roots.

MIPs constitute channels for small polar molecules across membranes. Since the preferred substrate usually is water, they are often referred to as aquaporins (AQPs). The 35 members of the AQP family found in Arabidopsis may be divided into four subfamilies, of which the PIP subfamily is the largest, with 13 members [18]. As indicated by the name, the main location of PIPs is expected to be the plasma membrane. PIPs are crucial for water uptake by the root system and for maintenance of water balance in the plant [19].

Peptides belonging to all 13 isoforms of the PIP subfamily were identified (Table 1). PIPs cluster into two subgroups, PIP1 and PIP2, with a sequence identity of about 70% between the subgroups and an identity within the subgroups of up to 96% in Arabidopsis [18]. Therefore, all PIP amino acid sequences were aligned to identify unique peptides produced by trypsin cleavage. This was necessary since the program used (Mascot) could not be trusted in this respect, but falsely identified unique peptides. Based on these alignments, nine PIP isoforms could be identified based on one or more unique peptides (Table 1, top).

**Figure 7:**
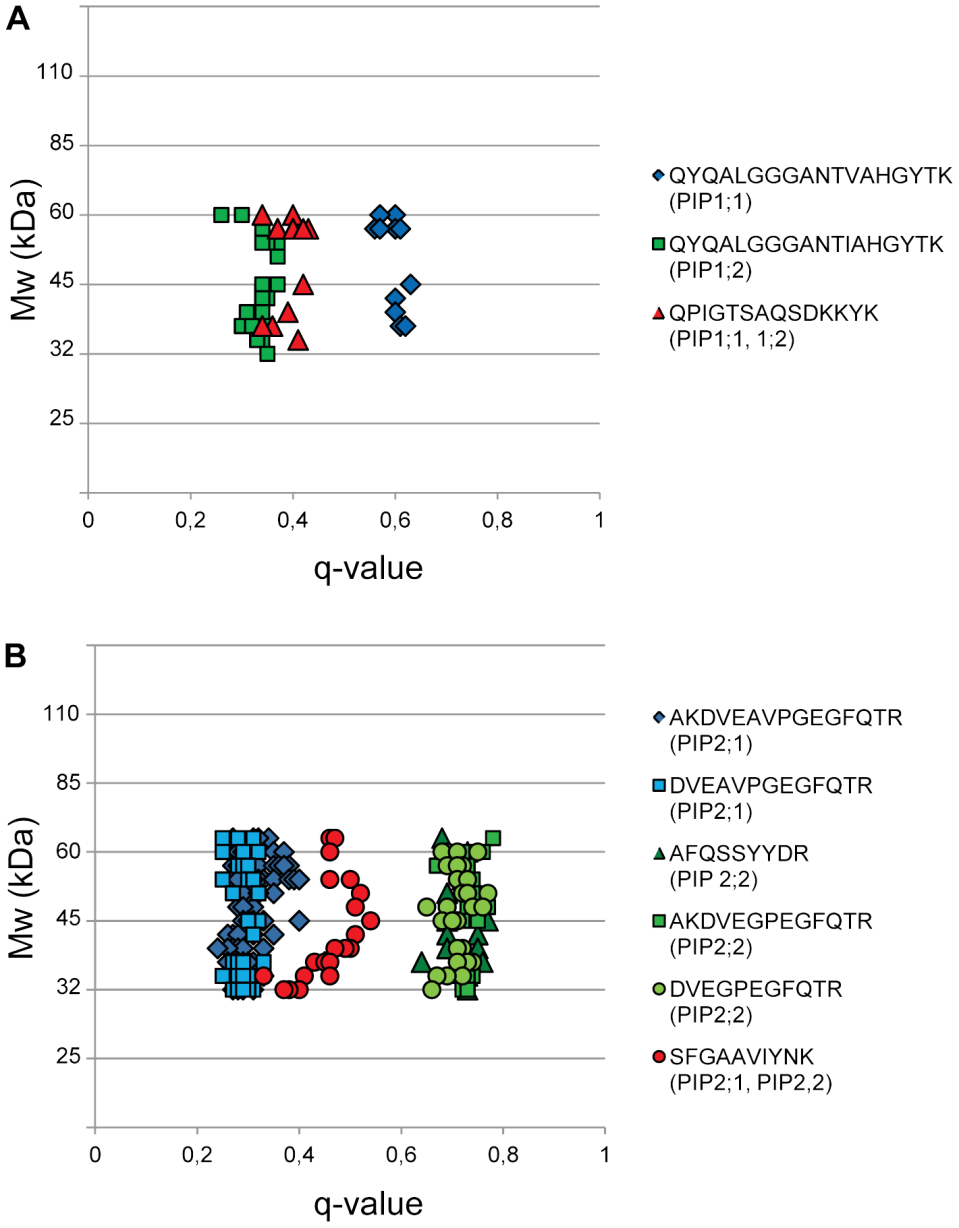
Q-values for peptides of PIP isoforms. This is simplified version of the plot mentioned in the Data processing part. A, Q-values for two peptides unique to PIP1;1 and PIP1;2, respectively, and for a peptide shared between these two isoforms. B, Q-values for two peptides unique to PIP2;1 and for three peptides unique to PIP2;2, and for a peptide shared between these two isoforms. Note that PIPs with a monomer molecular mass of about 30 kDa band upon SDS-PAGE both as monomers and dimers, and also in the area in between 30 and 60 kDa. The molecular weights on the y axis are those indicated by the Mw standards used in the SDS-PAGE; the scale is therefore not lineal.

PIPs are major proteins of plant plasma membranes [20] and band upon SDS-PAGE both as monomers at about 30 kDa and as dimers at about 60 kDa (e.g., [21]). Indeed, peptides originating from PIPs were identified in gel segments corresponding to both 30 and 60 kDa, as well as to the area in between (Figure 7). Figure 7A shows data for a peptide unique to PIP1;1 with an average q-value of 0.60, and for a peptide unique to PIP1;2 with an average q-value of 0.34. PIP1;1 is thus present at higher abundance in root tissue compared to leaf tissue, whereas the opposite is true for PIP1;2. Data are also shown for a peptide shared between PIP1;1 and PIP1;2 with an average q-value of 0.39. At equal amounts of the two isoforms we would expect a q-value of 0.47 for this shared peptide. The q-value of 0.39 suggests that the overall abundance in the plant plasma membranes of PIP1;2 is approximately four times higher than that of PIP1;1. Figure 7 B shows data for two peptides unique to PIP2;1 with q-values of 0.30 and 0.29, respectively, and three peptides unique to PIP2;2 with q-values of 0.74, 0.73 and 0.71, respectively, showing PIP2;1 to be more distributed to leaf tissue and PIP2;2 to be more distributed to root tissue. Figure 7 also demonstrates the very good agreement between different data points for one specific peptide (A and B) as well as for data obtained for different unique peptides of a protein (B). One peptide shared between PIP2;1 and PIP2;2 was also detected with an average q-value of 0.46, showing PIP2;1 and PIP2;2 to be present in about equal amounts (Figure 7B). Similarly, based on peptides shared between PIP2;1 and PIP2;4 and between PIP2;2 and PIP2;3 (Table 1), the ratios of abundance for these two pairs can be calculated to about 10/1. The proportions of isoforms based on shared peptides agree well with published mRNA data [21] for three of the pairs, PIP2;1/2;2, PIP2;1/2;4 and PIP2;2/2;3, but not for the PIP1;1/1;2 pair.

Four of the PIPs showed a tissue-specific localization: PIP1;5, 2;3, and 2;4 were only found in root tissue, whereas PIP2;6 was only found in leaf tissue. A peptide (VGANKFPER; Table 1), shared between all members of the PIP1 subfamily, with a q-value of 0.41 suggests that the PIP1 subfamily is 50% more abundant in leaf tissue than in root tissue. There is a fair agreement between our q-values for the different PIP isoforms and the mRNA data in Alexandersson et al. [21]. These mRNA data should be particularly relevant as a comparison since they were obtained with Arabidopsis grown in a similar way to our plants and using a non-commercial microarray with probes carefully designed to minimize overlap between isoforms. However, there is also a fair agreement with the mRNA-data in Genevestigator [22] (Table 1). A good agreement with mRNA levels should not always be expected, not least because some PIP isoforms are also present in other membranes than the plasma membrane, e.g., due to internalization and recycling of PIPs [19].

In addition to the nine PIP isoforms, another 32 proteins were identified with unique peptides (Table 2, Table S2). These have all previously been suggested to be localized to the plasma membrane (TAIR and/or references below) except for the four “unknowns” for which there is less information. These includes TIP(tonoplast intrinsic protein)1;2, which has also been identified in previous proteomic studies of Arabidopsis leaf [12] and root [23] plasma membranes.

**Table 2:** Integral plasma membrane proteins detected by MS and their relative abundance in leaf and root tissue (q-value).

AGI Code	Name	TAIR Description	MW	TM^a^	Uniq Pept^b^	Q-value (SD)^c^	No. of Spec^d^	GV^e^
***Transporters***								
AT3G61430	PIP1;1	Aquaporin	30897	5	1	0.60	12	lR
AT2G45960	PIP1;2	Aquaporin	30806	5	1	0.34	23	LR
AT4G23400	PIP1;5	Aquaporin	30855	5	1	1.00	2	LR
AT3G53420	PIP2;1	Aquaporin	30474	6	2	0.30 (0.01)	151	Lr
AT2G37170	PIP2;2	Aquaporin	30662	6	3	0.73 (0.01)	96	lR
AT2G37180	PIP2;3	Aquaporin	30638	6	1	1.00	2	lR
AT5G60660	PIP2;4	Aquaporin	31217	6	2	1.00 (0.00)	59	R
AT2G39010	PIP2;6	Aquaporin	31258	7	2	0.00 (0.00)	13	L
AT4G35100	PIP2;7	Aquaporin	29742	5	1	0.37	15	LR
AT3G26520	TIP1;2	Aquaporin	25889	7	1	0.70	20	LR
AT1G59870	ABCG36	ABC transporter	165831	15	3	0.16 (0.14)	14	Lr
AT3G47960		Oligopeptide transporter, H^+^ symport	67778	12	1	0.84	23	L
AT2G18960	AHA1	Plasma membrane H^+^-ATPase	104614	10	1	0.44	1	LR
***Membrane trafficking***								
AT3G09740	SYP71*	Syntaxin, Qc-SNARE	30135	1	2	0.45 (0.04)	9	LR
ATG09740	SYP71*	Syntaxin, Qc-SNARE	30135	1	2	0.73 (0.00)	7	LR
AT3G09740	SYP71*	Syntaxin, Qc-SNARE	30135	1	2	0.00 (0.00)	4	LR
AT3G11820	SYP121	Syntaxin, Qa-SNARE	38105	1	2	0.39 (0.02)	6	LR
AT5G08080	SYP132	Syntaxin, Qa-SNARE	34225	1	3	0.57 (0.01)	7	lR
AT2G20990	SYT1	Synaptotagmin	61933	1	1	0.51	4	LR
AT1G61250	SC3	Secretory carrier	32763	4	2	0.49 (0.01)	6	LR
***Cell-wall related proteins***								
AT2G04780	FLA7	Fasciclin-like arabinogalactan-protein	26845	1	1	0.53	15	lR
AT2G45470	FLA8	Fasciclin-like arabinogalactan-protein	43162	1	2	0.00 (0.00)	20	LR
AT4G12420	SKU5	Cu^2+^ binding, root tip growth	65767	1	2	0.30 (0.01)	6	LR
AT2G44790	UCC2	Uclacyanin, blue copper protein	20512	1	2	1.00 (0.00)	43	R
AT4G26690	SHV3	Glycerophosphoryldiester phosphodiesterase	82967	1	1	1.00	1	LR
AT5G55480	SVL1	Glycerophosphoryldiester phosphodiesterase	84511	1	2	0.47 (0.08)	2	lR
AT1G66970	SVL2	Glycerophosphoryldiester phosphodiesterase	84192	1	1	0.00	8	L
AT3G04010		O-Glycosyl hydrolase, family 17	54482	1	1	0.49	5	R
***Signal transduction and stress responses***								
AT3G08510	PLC2	Phosphoinositide-spec phospholipase C	66122	2	10	0.42 (0.15)	53	Lr
AT3G19820	DWF1	Brassinosteroid biosynthesis	65394	1	2	0.70 (0.00)	4	lR
AT3G48890	MSBP2	Progesterone binding protein	25367	1	1	0.40	3	LR
AT5G06320	NHL3	Similar to hairpin-induced (tobacco)	26444	1	1	0.00	1	Lr
AT1G30360	ERD4	Early responsive to dehydration	82282	11	9	0.37 (0.03)	49	LR
AT1G63500		Protein kinase	55504	1	3	0.59 (0.11)	7	LR
AT3G51330		Aspartyl protease	58625	1	1	0.82	1	lR
AT4G04720	CPK21	Ca^2+^-dependant protein kinase	60199	1	1	0.60	3	Lr
AT5G53560	B5-A	Cytochrome b5	15132	1	2	0.51 (0.00)	12	lR
AT2G37710	LRK1	L-Lectin (LEC) RLK	75779	1	1	1.00	6	Lr
AT3G02880		LRR III (5) RLK	68167	2	2	0.63 (0.01)	2	LR
***Unknown***								
AT1G58270	ZW9	Unknown molecular functions	45235	1	1	1.00	2	lR
AT2G39530		Unknown molecular functions	19191	3	1	1.00	13	R
AT4G15610		Unknown molecular functions	20796	4	1	0.81	12	lR
AT5G44550		Unknown molecular functions	21019	4	1	1.00	7	R

Proteins are grouped according to function and all annotation is via the database TAIR.

a Predicted transmembrane domains determined by Phobius (Kall et al., 2004).

b Number of unique peptides first identified by MS/MS and then detected in reliable ^14^N/^15^N spectra used for determination of the q-value for that specific protein. All these spectra have fulfilled all criteria for that specific peptide/protein, including correct position of the protein on the SDS-gel, consistent elution of the peptide in the nano-LC gradient, and correct peptide molecular mass as well as isotopic pattern.

c The q-value is a measure of the distribution of the peptide between root and leaf plasma membranes: A q-value of 1 means that the peptide is found in roots only; 0, in leaves only. SD is the standard deviation for the q-value of the protein based on all unique peptides for that specific protein.

d Number of MS spectra containing reliable data for the ^14^N/^15^N peptide pair(s) used to determine the q-value.

e Genevestigator data for mRNA distribution between Arabidopsis leaf rosettes and roots converted to a simple letter code: L, mRNA found in leaves only, R, in roots only; LR, about equally distributed between leaves and roots; Lr, mainly in leaves; lR, mainly in roots.

* The three q-values for SYP71 were obtained with two unique peptides found in three neighboring segments of the SDS gel (see text for discussion).

Thirteen transporters were identified with unique peptides: the nine PIPs discussed above (Table 1), one TIP, one ATP-binding cassette (ABC) transporter, one oligopeptide transporter and one isoform of the plasma membrane H^+^-ATPase family. The plasma membrane H^+^-ATPase constitutes several percent of total membrane protein [24] and creates the H^+^ and electrical gradient across the plasma membrane which drives secondary active transport. The H^+^-ATPase in Arabidopsis is encoded by a gene family with 12 members producing 11 expressed isoforms (AHA1-11) with an amino acid sequence identity of 64 to 91% [9]. Eleven AHA peptides were identified, however only one of these was unique and belongs to AHA1 (Table 2, Table S2). The q-value of this peptide was 0.44, and the q-values for the other 10 peptides, shared between two to eight of the expressed 11 isoforms, ranged between 0.36 and 0.80 with a mean value of 0.59 (data not shown). This suggests that the H^+^-ATPase family as a whole is about equally expressed in leaves and roots, in agreement with the immunostaining in Figure 1. The ABC-transporter identified with three unique peptides (Table S2) was the highly expressed PEN3/PDR8 (ABCG36), which is known to be highly expressed and confers resistance to a number of pathogens, in particular of the leaf, and is suggested to extrude compounds toxic to the pathogens at the invasion site [25]. However, PEN3/PDR8 seems to transport a broad range of compounds, including heavy metal ions such as Cd^2+^ and Pb^2+^, and is more abundant in shoots than in roots of 2-week-old Arabidopsis seedlings as demonstrated by immunostaining [26], which is in good agreement with our q-value of 0.16.

Five proteins involved in vesicle transport at the plasma membrane were identified with q-values suggesting that they all are expressed in both leaves and roots, in agreement with literature data (see below), although our data for SYP71, a Qc-SNARE [27], are more complex. We detected SYP71 with two unique peptides (Table S2) in three neighboring segments of the gel, with different q-values for each segment, suggesting the presence of three variants of the protein migrating to slightly different positions in the gel. This could be due to different posttranslational modifications and/or to expression of different splice variants. creating some degree of tissue specificity for the ubiquitously expressed SYP71. SYP71 is located in both the endoplasmic reticulum and the plasma membrane and is expressed in all vegetative tissues, as demonstrated by both staining with ß-glucuronidase (GUS) and immunostaining [27]. The *Lotus japonicus* SYP71 homolog was recently implicated in symbiotic nitrogen fixation and shown to be expressed in both shoots, roots and nodules [28]. A ubiquitous expression is typical for the SNARE proteins in Arabidopsis as shown by reversed transcription-PCR (RT-PCR) [29] and confirmed for the Qa-SNARES SYP121 and 132 using GFP-fusion proteins [30]. A ubiquitous expression of plant SNAREs is supported by a recent proteomic study on plasma membranes isolated from leaves, xylem and phloem from poplar trees [11]; in the poplar study, none of the 13 identified SNAREs was tissue specific, and 10 of the 13 SNAREs were detected in all three tissues investigated. SYT1, a synaptotagmin, is involved in plasma membrane repair where resealing of membrane disruptions requires exocytotic addition of internal membrane, and is found in all tissues as shown by GUS staining [31].

Of the eight proteins classified as “cell wall-related”, previous organ/tissue localizations exist for both of the fasciclin-like arabinogalactan proteins, FLA7 and 8, for one of the copper proteins, SKU5, and for all three glycerophosphodiester phosphodiesterases, SHV3 and SVL1 and 2 (SHV3-like 1 and 2). FLAs have been suggested to affect cellulose deposition [32] and to be involved in responses to abiotic stress [33]. RNA gel blots suggest that FLA8 is about equally expressed in leaves and roots of 14-day-old Arabidopsis seedlings [33] which is in direct contradiction to our data, which suggest FLA8 to be mainly expressed in leaves. However, a compilation of expressed sequence tag (EST) data suggests that FLA8 has a higher expression in “above ground organs” than in roots and that the opposite is true for FLA7 [33], which agrees better with our q-values of 0.53 and 0 for FLA7 and 8, respectively. SKU5 is suggested to have a role in cell wall expansion, and immunostaining indicates that it is present in both roots and leaves, particularly in expanding tissues [34], in fair agreement with our data. SHV3 and its homologs SVL1 and 2 are thought to be involved in primary cell wall organization [35]. Using RT-PCR, SHV3 was found to be expressed in both roots and leaves of 6-week-old Arabidopsis seedlings, whereas SVL1 showed expression in roots only, and SVL2 in leaves only. However, using GUS staining of promoter activity in 7-day-old seedlings, contradictory results were obtained for SHV3 and SVL1; SHV3 was found to be expressed in roots only, SVL1 in both roots and leaves, and SVL2, as also indicated by RT-PCR, in leaves only [35], all in very good agreement with our data.

Eleven proteins related to signal transduction and stress responses were identified, of which only three, PLC2, MSBP2 and NHL3, have previously been localized to a tissue or organ in Arabidopsis. The phosphoinositide-specific phospholipase C, PLC2, which produces the two second messengers inositol-1,4,5-trisphosphate and diacylglycerol, is expressed about equally in roots and leaves of 1 to 2-week-old Arabidopsis seedlings according to RNA blots [36], in good agreement with our q-value of 0.42. DWARF1 (DWF1) catalyzes an early step in the biosynthesis of the signal molecule brassinosteroid [37]. There are no localization data for the Arabidopsis protein but the single maize homolog ZmDWF1 was shown to be expressed in all tissues examined, with a relatively high expression in roots, particularly in young, actively developing roots [38], which compares well with our q-value of 0.70. The progesterone binding protein, MSBP2 (membrane steroid binding protein 2), is expressed in rosette leaves of Arabidopsis [39] as determined with RNA blots; however, roots were not investigated. We found MSBP2 about equally distributed between roots and leaves. The *NHL3* gene belongs to a family of *NDR1/HIN1*-like (*NHL*) genes that are homologs of the nonrace-specific disease resistance (*NDR1*) and harpin-induced (*HIN1*) genes in *Nicotiana tabacum* [40]. Overexpression of the NHL3 protein in Arabidopsis correlates with increased resistance to the pathogen *Pseudomonas syringae* [41] and the authors demonstrate expression in leaves by immunoblotting. Roots were not investigated, however, but we find the protein in leaves only.

The four proteins with “unknown molecular functions” (TAIR) were all mainly found in roots and may be involved in stress responses. The ZW9 protein has a TRAF (tumor necrosis factor receptor–associated factor) motif. TRAF-proteins in mammals link cell-surface receptors to intracellular signaling pathways in innate immune responses [42]. The plant innate immune system is well developed and numerous receptors (RLKs) are found in plasma membranes (e.g, 10, Table S1). TRAF domain-containing genes were also recently linked to virus infection in Arabidopsis by association mapping [43]. The other three “unknowns” are all members of the same plant-specific “uncharacterized protein family” (UPF0497, TAIR), and have molecular masses of about 20 kDa. Genome-wide analysis of gene expression in Arabidopsis have associated members of this protein family with responses to both abiotic [44] and biotic [45] stress.

## Conclusions

Only 41 of the 188 integral proteins identified by the software Mascot (Table S1) generated reliable ^14^N/^15^N peptide spectra and were identified with at least one unique peptide (Table 2, Table S2), whereas the remaining 147 proteins were excluded due to unreliable peptide spectra, or, most commonly, because they were only identified with peptides shared by other proteins. This latter problem arises due to the many gene families in Arabidopsis resulting in protein isoforms with high amino acid sequence identity. The problem is increased because the shared peptides will be the most abundant ones and thus most easily be detected in the mass spectrometer. Notably, however, peptides shared between isoforms may also be useful and give information on the proportions of isoforms, or on the relative abundance of a whole protein family, as demonstrated with the PIP aquaporins. To improve detection of unique peptides, a library with unique peptides of all proteins of interest could be constructed beforehand and the corresponding masses searched for, as was done here for the PIP aquaporin subfamily. A similar targeted search for PIP subfamily members in total membrane fractions from Arabidopsis leaves and roots, respectively, identified nine PIP isoforms [46], of which eight coincide with the nine PIPs found in our study on purified plasma membranes. One result of the many isoforms in multicellular organisms is tissue specific expression. We found that 14 of the 41 uniquely identified proteins (i.e. 34%), including four of the nine PIPs, had an extreme distribution to either leaf or root tissue, whereas most proteins were found to be more equally distributed between the two tissues. This agrees well with a recent proteomic study on plasma membranes from leaves, xylem, and cambium/phloem from poplar trees [11], where 44% of the integral proteins were detected in one tissue only.

A comparison between our data for protein levels and corresponding data for mRNA levels in the widely used database Genevestigator [22] shows agreement for only about two thirds of the proteins. However, localization data available in the literature for 21 of the 41 proteins show a better agreement with our data, in particular data based on immunostaining of proteins and GUS-staining of promoter activity. The reason for the lower agreement with mRNA data could be problems with probe specificity in the microarrays used, whereas antibodies raised against peptides as well as promoters can be made more isoform-specific. Moreover, a good correlation between protein and mRNA abundance should not always be expected for several reasons; e.g., regulation of protein expression occurs both at the transcriptional and translational level, which makes it difficult to predict the proteome based on mRNA abundance [47]. In addition, the rate of protein turnover will affect the final protein level. Thus, detection and quantification of isoform-specific peptides by proteomics should generate the most reliable data for the proteome, provided that the protocol used is carefully designed. Not least important is the software used for selection of reliable spectra, together with other criteria for identification of the specific peptide/protein. In the present work, our software and additional criteria reduced the dataset from more than 30,000 spectra to about 1000 spectra to be subjected to manual inspection, which we still consider a necessary final step in order to obtain results of high quality.

## Supporting Information

Table S1 **Integral membrane proteins detected by MS/MS in Arabidopsis leaf and root plasma membranes.**
(DOCX)

Table S2 **Integral membrane proteins and corresponding unique peptides detected by mass spectrometry in Arabidopsis plasma membranes and the relative abundance in leaf and root tissue (Q-value).**
(DOCX)
