# Supplementary material for: Relative Abundance of Integral Plasma Membrane Proteins in Arabidopsis Leaf and Root Tissue Determined by Metabolic Labeling and Mass Spectrometry
Source: PLoS One. 2013 Aug 19;8(8):e71206. doi: 10.1371/journal.pone.0071206 (PMC3747180; doi:10.1371/journal.pone.0071206)
Supplement: Table S1 — Integral membrane proteins detected by MS/MS in Arabidopsis leaf and root plasma membranes. (DOCX) [file pone.0071206.s001.docx]

| **Table S1** Integral membrane proteins detected by MS/MS in Arabidopsis leaf and root plasma membranes  The proteins are grouped according to function and all annotation is via the database TAIR | | | | | |
| --- | --- | --- | --- | --- | --- |
| **AGI Code** | **Name** | **TAIR description** | **MW** | **TM^a^** | **Number of Peptides^b^** |
| ***Transporters*** | | | | | |
| AT2G18960 | AHA1 | Plasma membrane H^+^-ATPase | 104614 | 10 | 11 |
| AT4G30190 | AHA2 | Plasma membrane H^+^-ATPase | 104734 | 10 | 2 |
| AT5G57350 | AHA3 | Plasma membrane H^+^-ATPase | 104897 | 10 | 2 |
| AT2G24520 | AHA5 | Plasma membrane H^+^-ATPase | 103224 | 10 | 2 |
| AT1G17260 | AHA10 | Plasma membrane H^+^-ATPase | 105262 | 10 | 2 |
| AT5G62670 | AHA11 | Plasma membrane H^+^-ATPase | 105398 | 10 | 11 |
| AT5G57110 | ACA8 | Ca^2+^-ATPase | 116728 | 8 | 4 |
| AT1G15690 | AVP1 | H^+^-pumping PPi-ase | 67706 | 13 | 2 |
| AT2G21410 | VHA-A2 | V-type H^+^-ATPase subunit | 93616 | 7 | 3 |
| AT4G39080 | VHA-A3 | V-type H^+^- ATPase subunit | 93344 | 6 | 7 |
| AT3G28380 | ABCB17 | ABC transporter | 136831 | 8 | 2 |
| AT3G62150 | ABCB21 | ABC transporter | 140066 | 11 | 4 |
| AT4G01820 | ABCB3 | ABC transporter | 133777 | 10 | 4 |
| AT2G47000 | ABCB4 | ABC transporter | 139397 | 10 | 3 |
| AT3G16340 | ABCG29 | ABC transporter | 160993 | 13 | 5 |
| AT2G29940 | ABCG31 | ABC transporter | 160511 | 14 | 3 |
| AT2G37280 | ABCG33 | ABC transporter | 161232 | 14 | 2 |
| AT1G59870 | ABCG36 | ABC transporter | 165831 | 15 | 16 |
| AT5G43350 | PHT1;1 | Phosphate transporter | 57616 | 12 | 1 |
| AT5G43370 | PHT1;2 | Phosphate transporter | 57949 | 12 | 1 |
| AT2G38940 | PHT1;4 | Phosphate transporter | 58903 | 11 | 3 |
| AT2G38290 | AMT2 | Ammonium transporter, high affinity | 50906 | 11 | 3 |
| AT5G26340 | STP13 | Hexose transporter, high affinity, H^+^ symport | 57781 | 12 | 2 |
| AT3G19930 | STP4 | Sucrose transporter, H^+^ symport | 57457 | 12 | 2 |
| AT1G71880 | SUC1 | Sucrose transporter, H^+^ symport | 55222 | 12 | 2 |
| AT4G08620 | SULTR1 | Sulfate transporter, high affinity | 70897 | 10 | 5 |
| AT1G78000 | SULTR1;2 | Sulfate transporter | 72060 | 10 | 3 |
| AT3G54140 | PTR1 | Oligopeptide transporter | 64620 | 10 | 2 |
| AT3G47960 |  | Oligopeptide transporter, H^+^ symport | 67778 | 12 | 3 |
| AT3G61430 | PIP1;1 | Aquaporin | 30897 | 5 | 4 |
| AT2G45960 | PIP1;2 | Aquaporin | 30806 | 5 | 5 |
| AT1G01620 | PIP1;3 | Aquaporin | 30841 | 5 | 1 |
| AT4G00430 | PIP1;4 | Aquaporin | 30901 | 5 | 5 |
| AT4G23400 | PIP1;5 | Aquaporin | 30855 | 5 | 3 |
| AT3G53420 | PIP2;1 | Aquaporin | 30683 | 6 | 2 |
| AT2G37170 | PIP2;2 | Aquaporin | 30662 | 6 | 6 |
| AT2G37180 | PIP2;3 | Aquaporin | 30638 | 6 | 4 |
| AT5G60660 | PIP2;4 | Aquaporin | 31217 | 6 | 2 |
| AT3G54820 | PIP2;5 | Aquaporin | 30798 | 6 | 2 |
| AT2G39010 | PIP2;6 | Aquaporin | 31258 | 7 | 3 |
| AT4G35100 | PIP2;7 | Aquaporin | 29952 | 5 | 3 |
| AT2G16850 | PIP2;8 | Aquaporin | 29709 | 5 | 2 |
| AT3G26520 | TIP1;2 | Aquaporin | 25889 | 7 | 1 |
| AT2G46430 | CNGC3 | Cyclic nucleotide gated channel | 82472 | 6 | 3 |
| AT4G18160 | TPK3 | Ca^2+^-activated, outward rectifying K^+^ channel | 49156 | 5 | 1 |
| ***Receptors*** | | | | | |
| AT3G09780 | CRR1 | CR4L RLK (Crinkly4 related 1) | 86184 | 1 | 1 |
| AT2G39180 | CRR2 | CR4L RLK | 85302 | 1 | 1 |
| AT3G51550 | FER | CrRLK1L-1 (K) RLK | 98829 | 1 | 3 |
| AT2G23200 |  | CrRLK1L-1 RLK | 93720 | 1 | 2 |
| AT1G70530 |  | DUF26 RLK | 72421 | 1 | 4 |
| AT4G23180 | CRK10 | DUF26 RLK | 75092 | 1 | 6 |
| AT4G23230 | CRK15 | DUF26 RLK | 56940 | 1 | 4 |
| AT4G23250 | CRK17 | DUF26 RLK | 113284 | 2 | 2 |
| AT4G23260 | CRK18 | DUF26 RLK | 73634 | 1 | 2 |
| AT4G23290 | CRK21 | DUF26 RLK | 67138 | 1 | 2 |
| AT4G23310 | CRK23 | DUF26 RLK | 93569 | 1 | 3 |
| AT4G11460 | CRK30 | DUF26 RLK | 78833 | 1 | 2 |
| AT4G11470 | CRK31 | DUF26 RLK | 75766 | 1 | 2 |
| AT4G11480 | CRK32 | DUF26 RLK | 74731 | 1 | 2 |
| AT5G56890 |  | Extensin (EXT) RLK | 119740 | 1 | 3 |
| AT2G37710 | LRK1 | L-Lectin (LEC) RLK, response to salicylic acid | 75779 | 1 | 6 |
| AT3G46400 |  | LRR I (1) RLK | 99947 | 1 | 2 |
| AT2G14510 |  | LRR I (2) RLK | 98314 | 1 | 2 |
| AT2G19210 |  | LRR I (2) RLK | 98999 | 1 | 2 |
| AT2G19230 |  | LRR I (2) RLK | 115558 | 1 | 2 |
| AT1G51850 |  | LRR I (3) RLK | 96858 | 1 | 3 |
| AT1G51890 |  | LRR I (3) RLK | 100346 | 1 | 3 |
| AT3G46370 |  | LRR I (3) RLK | 88807 | 1 | 2 |
| AT2G14440 |  | LRR I (4) RLK | 100398 | 1 | 2 |
| AT5G16590 | LRR1 | LRR III (5) RLK | 67991 | 1 | 2 |
| AT2G26730 |  | LRR III (5) RLK | 72106 | 1 | 2 |
| AT3G02880 |  | LRR III (5) RLK | 68167 | 2 | 9 |
| AT1G51805 |  | LRR RLK | 98755 | 1 | 5 |
| AT1G56145 |  | LRR RLK | 112576 | 1 | 3 |
| AT3G46330 | MEE39 | LRR RLK, embryo development ending | 98677 | 1 | 2 |
| AT5G49760 |  | LRR VIII-1 (9) RLK | 105383 | 1 | 4 |
| AT1G56140 |  | LRR VIII-2 (10) RLK | 113753 | 1 | 2 |
| AT1G53430 |  | LRR VIII-2 (6) RLK | 113976 | 1 | 9 |
| AT1G53420 |  | LRR VIII-2 (8) RLK | 108106 | 1 | 3 |
| AT1G53440 |  | LRR VIII-2 (9) RLK | 115748 | 1 | 3 |
| AT1G56130 |  | LRR VIII-2 (9) RLK | 113883 | 1 | 2 |
| AT3G14840 |  | LRR VIII-2 (9) RLK | 112748 | 1 | 6 |
| AT1G06840 |  | LRR VIII-I (11) RLK | 104589 | 1 | 1 |
| AT3G28450 |  | LRR X (5) RLK | 67814 | 1 | 4 |
| AT4G28650 |  | LRR XI (22) RLK | 111264 | 1 | 2 |
| AT5G07280 | EMS1 | LRR-RLK | 130858 | 1 | 1 |
| AT2G45590 |  | RLCK XI RLK | 75554 | 1 | 1 |
| AT1G65790 | ARK1 | SD-1 RLK | 97025 | 1 | 2 |
| AT1G11340 |  | SD-1 RLK | 103922 | 1 | 2 |
| AT1G11410 |  | SD-1 RLK | 95862 | 1 | 2 |
| AT1G61360 |  | SD-1 RLK | 90933 | 1 | 2 |
| AT1G11350 | CBRLK1 | SD-1 RLK, calmodulin-binding | 94372 | 1 | 2 |
| AT2G42800 | RLP29 | Receptor like protein | 51224 | 1 | 3 |
| ***Cell wall-related proteins*** | | | | | |
| AT5G05170 | CESA3 | Cellulose synthase, primary cell wall | 121032 | 8 | 2 |
| AT4G03550 | GSL5 | Callose synthase | 207464 | 16 | 2 |
| AT3G59100 | GSL11 | Callose synthase like | 225562 | 17 | 3 |
| AT3G07160 | GSL10 | 1,3-beta-D-glucan synthase | 223929 | 15 | 7 |
| AT2G36850 | GSL8 | 1,3-beta-D-glucan synthase | 219657 | 13 | 2 |
| AT1G02850 |  | Beta glucosidase | 52581 | 1 | 1 |
| AT3G04010 |  | O-Glycosyl hydrolase, family 17 | 54482 | 1 | 5 |
| AT4G31140 |  | O-Glycosyl hydrolase, family 17 | 53081 | 1 | 4 |
| AT2G04780 | FLA7 | Fasciclin-like arabinogalactan-protein | 26886 | 1 | 2 |
| AT2G45470 | FLA8 | Fasciclin-like arabinogalactan-protein | 43162 | 1 | 2 |
| AT1G03870 | FLA9 | Fasciclin-like arabinogalactan-protein | 26115 | 1 | 1 |
| ***Membrane trafficking*** | | | | | |
| AT1G64090 | RTNLB3 | Reticulon family protein | 28735 | 3 | 2 |
| AT3G10260 |  | Reticulon family protein \| | 28122 | 3 | 2 |
| AT1G48240 | NPSN12 | Novel plant SNARE 12 | 29887 | 1 | 2 |
| AT3G17440 | NPSN13 | Novel plant SNARE 13 | 30566 | 1 | 2 |
| AT3G13870 | RHD3 | GTP-binding protein | 89091 | 3 | 3 |
| AT1G61250 | SC3 | Secretory carrier | 32763 | 4 | 3 |
| AT1G78720 | Sec61 | Protein transport | 52440 | 10 | 2 |
| AT2G20990 | SYTA | Synaptotagmin | 61933 | 1 | 6 |
| AT4G32150 | VAMP711 | Vesicle transport, v-SNARE (Synaptobrevin-like) | 25251 | 1 | 2 |
| AT1G04750 | VAMP721 | Vesicle-mediated transport (Synaptobrevin) | 20722 | 1 | 4 |
| AT2G33120 | VAMP722 | SAR1, Synaptobrevin-like protein | 24928 | 1 | 4 |
| AT2G32670 | VAMP725 | Vesicle-mediated transport (Synaptobrevin) | 32691 | 2 | 1 |
| AT1G04760 | VAMP726 | Synaptobrevin-like protein | 24873 | 1 | 1 |
| AT3G09740 | SYP71 | Syntaxin, Qc-SNARE | 30135 | 1 | 4 |
| AT3G11820 | SYP121 | Syntaxin | 38105 | 1 | 3 |
| AT3G52400 | SYP122 | Syntaxin | 38042 | 1 | 1 |
| AT5G08080 | SYP132 | Syntaxin, SNAP receptor activity | 34225 | 1 | 3 |
| AT5G42570 |  | Intracellular protein transport? | 24549 | 3 | 2 |
| ***Others*** | | | | | |
| AT1G63500 |  | Protein kinase | 55504 | 1 | 5 |
| AT3G20410 | CPK9 | Ca^2+^-dependant protein kinase | 60724 | 1 | 8 |
| AT4G21940 | CPK15 | Ca^2+^-dependant protein kinase | 62575 | 1 | 2 |
| AT5G12180 | CPK17 | Ca^2+^-dependant protein kinase | 58484 | 1 | 1 |
| AT4G04720 | CPK21 | Ca^2+^-dependant protein kinase | 60199 | 1 | 2 |
| AT1G76040 | CPK29 | Ca^2+^-dependent protein kinase | 37195 | 2 | 1 |
| AT1G50700 | CPK33 | Ca^2+^-dependent protein kinase | 58968 | 1 | 4 |
| AT5G19360 | CPK34 | Ca^2+^-dependant protein kinase | 58593 | 1 | 1 |
| AT5G53560 | B5-A | Cytochrome b5 | 15132 | 1 | 2 |
| AT2G14100 | CYP705A13 | Cytochrome P450 | 59404 | 2 | 2 |
| AT3G20140 | CYP705A23 | Cytochrome P450 | 58058 | 4 | 3 |
| AT4G22690 | CYP706A1 | Cytochrome P450 | 63248 | 1 | 4 |
| AT4G22710 | CYP706A2 | Cytochrome P450 | 59425 | 1 | 4 |
| AT5G48000 | CYP708A2 | Cytochrome P450 | 58877 | 2 | 1 |
| AT3G26170 | CYP71B19 | Cytochrome P450 | 57518 | 1 | 1 |
| AT3G26180 | CYP71B20 | Cytochrome P450 | 57892 | 1 | 1 |
| AT1G13100 | CYP71B29 | Cytochrome P450 | 56312 | 2 | 2 |
| AT3G26330 | CYP71B37 | Cytochrome P450 | 57206 | 1 | 1 |
| AT2G02580 | CYP71B9 | Cytochrome P450 | 57084 | 1 | 1 |
| AT3G08510 | PLC2 | Phosphoinositide-specific phospholipase C | 66122 | 2 | 2 |
| AT5G04040 | SDP1 | Triacyl glycerol lipase | 92775 | 5 | 2 |
| AT3G14360 |  | Triacyl glycerol lipase | 59854 | 1 | 1 |
| AT5G55480 | SVL1 | Glycerophosphoryl diester phosphodiesterase | 84511 | 1 | 5 |
| AT1G66970 | SVL2 | Glycerophosphodiester phosphodiesterase | 84192 | 1 | 5 |
| AT4G26690 | SHV3 | Glycerophosphoryl diester phosphodiesterase | 82967 | 1 | 7 |
| AT4G00450 | CRP | Regulation of radial pattern formation | 251794 | 4 | 2 |
| AT3G19820 | DWF1 | Brassinosteroid biosynthesis | 65637 | 1 | 2 |
| AT4G04340 | ERD | Early-responsive to dehydration | 87893 | 9 | 5 |
| AT1G30360 | ERD4 | Early responsive to dehydration | 82282 | 11 | 4 |
| AT4G22120 |  | Early-responsive to dehydration | 87893 | 10 | 3 |
| AT3G23300 |  | Dehydration-responsive protein | 70110 | 1 | 2 |
| AT1G27950 | LTPG1 | Lipid transfer protein (GPI-anchor) | 20202 | 1 | 1 |
| AT3G48890 | MP2 | Progesterone binding protein | 25367 | 1 | 1 |
| AT5G06320 | NHL3 | Similar to hairpin-induced (tobacco) | 26444 | 1 | 3 |
| AT3G53520 | UXS1 | UDP-glucuronic acid decarboxylase | 48543 | 1 | 2 |
| AT4G12420 | SKU5 | Cu^2+^ binding, root tip growth | 65767 | 1 | 8 |
| AT2G44790 | UCC2 | Uclacyanin, blue copper protein | 20512 | 1 | 2 |
| AT1G03370 |  | Protein binding / zinc ion binding | 209163 | 1 | 4 |
| AT1G52200 |  | Response to oxidative stress | 21678 | 1 | 1 |
| AT1G52600 |  | Signal peptidase, putative | 20066 | 3 | 2 |
| AT1G65820 |  | Glutathione S-transferase | 16586 | 3 | 3 |
| AT4G27520 |  | Electron carrier, plastocyanin-like domain | 35156 | 1 | 3 |
| AT5G15350 |  | Electron carrier, plastocyanin-like domain | 19536 | 1 | 1 |
| AT5G35735 |  | Auxin-responsive family | 44011 | 5 | 1 |
| AT1G73650 |  | Oxidoreductase, acting on the CH-CH, lipid Metabolism | 33095 | 7 | 1 |
| AT3G51330 |  | Aspartyl protease | 58625 | 1 | 2 |
| AT1G78880 |  | Protease? | 50472 | 2 | 1 |
| AT3G19340 |  | Aminopeptidase? | 57151 | 2 | 3 |
| ***Unknown*** | | | | | |
| AT2G23810 | TET8 | Unknown molecular functions | 31550 | 4 | 1 |
| AT2G17120 | LYM2 | Unknown molecular functions (GPI-anchored) | 38855 | 1 | 2 |
| AT3G45600 | TET3 | Unknown molecular functions | 32494 | 4 | 3 |
| AT1G58270 | ZW9 | Unknown molecular functions | 45235 | 1 | 1 |
| AT1G65985 |  | Unknown molecular functions | 53500 | 1 | 2 |
| AT2G39530 |  | Unknown molecular functions | 19191 | 3 | 2 |
| AT3G06035 |  | Unknown molecular functions | 22381 | 1 | 2 |
| AT3G06390 |  | Unknown molecular functions | 21314 | 4 | 4 |
| AT3G27390 |  | Unknown molecular functions | 66139 | 6 | 2 |
| AT4G15610 |  | Unknown molecular functions | 20796 | 4 | 2 |
| AT4G20390 |  | Unknown molecular functions | 21249 | 4 | 1 |
| AT4G28770 |  | Unknown molecular functions | 31413 | 4 | 1 |
| AT5G17190 |  | Unknown molecular functions | 15046 | 3 | 1 |
| AT5G19230 |  | Unknown molecular functions | 20840 | 1 | 1 |
| AT5G44550 |  | Unknown molecular functions | 21019 | 4 | 1 |
| AT5G52420 |  | Unknown molecular functions | 26777 | 5 | 2 |
| AT1G05500 | SYTE | Unknown molecular functions | 63117 | 1 | 4 |
| AT1G11960 |  | Unknown molecular functions | 88452 | 3 | 2 |

^a^Predicted transmembrane domains determined by Phobius (Kall et al., 2004). ^b^Number of peptides identified with MS/MS for each protein (total ionscore ≥ 99.5 %).
