# Supplementary material for: Relative Abundance of Integral Plasma Membrane Proteins in Arabidopsis Leaf and Root Tissue Determined by Metabolic Labeling and Mass Spectrometry
Source: PLoS One. 2013 Aug 19;8(8):e71206. doi: 10.1371/journal.pone.0071206 (PMC3747180; doi:10.1371/journal.pone.0071206)
Supplement: Table S2 — Integral membrane proteins and corresponding unique peptides detected by mass spectrometry in Arabidopsis plasma membranes and the relative abundance in leaf and root tissue (Q-value). (DOCX) [file pone.0071206.s002.docx]

| **Table S2** Integral membrane proteins and corresponding unique peptides detected by mass spectrometry in Arabidopsis plasma membranes and the relative abundance in leaf and root tissue (Q-value)  The proteins are grouped according to function and all annotation is via the database TAIR | | | | | | |  |
| --- | --- | --- | --- | --- | --- | --- | --- |
| **AGI Code** | | **Name** | **TAIR description** | | **Unique Peptides^a^** | **Q-value (SD)^b^** | **Number of spectra^c^** |
|  | |  |  | |  |  |  |
| ***Transporters*** | | | | | | | |
| AT3G61430 | | PIP1;1 | | Aquaporin | QYQALGGGANTVAHGYTK | 0,60 (0,022) | 12 |
| AT2G45960 | | PIP1;2 | | Aquaporin | QYQALGGGANTIAHGYTK | 0,34 (0,025) | 23 |
| AT4G23400 | | PIP1;5 | | Aquaporin | QPIGTAAQTESK | 1,00 (0,000) | 2 |
| AT3G53420 | | PIP2;1 | | Aquaporin | AKDVEAVPGEGFQTR | 0,30 (0,036) | 104 |
|  | |  | |  | DVEAVPGEGFQTR | 0,27 (0,081) | 51 |
| AT2G37170 | | PIP2;2 | | Aquaporin | AFQSSYYDR | 0,74 (0,019) | 8 |
|  | |  | |  | AKDVEGPEGFQTR | 0,73 (0,018) | 48 |
|  | |  | |  | DVEGPEGFQTR | 0,71 (0,026) | 39 |
| AT2G37180 | | PIP2;3 | | Aquaporin | AKDVEGPDGFQTR | 1,00 (0,000) | 2 |
| AT5G60660 | | PIP2;4 | | Aquaporin | ALGSFGSFGSFR | 1,00 (0,000) | 4 |
|  | |  | |  | DLDVNESGPPAAR | 1,00 (0,000) | 55 |
| AT2G39010 | | PIP2;6 | | Aquaporin | SFGAAVIYNNQK | 0,00 (0,000) | 6 |
|  | |  | |  | VFQSTYYNR | 0,00 (0,000) | 7 |
| AT4G35100 | | PIP2;7 | | Aquaporin | TPYNTLGGGANTVADGYSK | 0,37 (0,037) | 15 |
| AT3G26520 | | TIP1;2 | | Aquaporin | NIAIGGVQEEVYHPNALR | 0,70 (0,019) | 20 |
| AT1G59870 | | ABCG36 | | ABC transporter | DISGVIKPGR | 0,24 (0,014) | 2 |
|  | |  | |  | NIEDIFSSGSR | 0,24 (0,021) | 2 |
|  | |  | |  | TLNFQTR | 0,00 (0,000) | 10 |
| AT3G47960 | |  | | Oligopeptide transporter, H^+^ symport | YTDQFR | 0,84 (0,024) | 23 |
| AT2G18960 | | AHA1 | | Plasma membrane H^+^-ATPase | IVIFGPNKLEEK | 0,44 | 1 |
|  | |  | |  |  |  |  |
| ***Membrane trafficking*** | | | | | | | |
| AT3G09740 | | SYP71 | Syntaxin, Qc-SNARE* | | EANISGDDAFAR | 0,00 (0,000) | 4 |
|  | |  |  | | EANISGDDAFAR | 0,42 (0,026) | 4 |
|  | |  |  | | EANISGDDAFAR | 0,73 (0,006) | 4 |
|  | |  |  | | NDLVLALPAR | 0,47 (0,030) | 5 |
|  | |  |  | | NDLVLALPAR | 0,73 (0,012) | 3 |
| AT3G11820 | | SYP121 | Syntaxin, Qa-SNARE | | VLDTINEIQER | 0,41 (0,014) | 2 |
|  | |  |  | | YFTVTGENPDER | 0,38 (0,031) | 4 |
| AT5G08080 | | SYP132 | Syntaxin, Qa-SNARE | | ENIQQEYR | 0,57 (0,008) | 5 |
|  | |  |  | | GSFELPR | 0,58 | 1 |
|  | |  |  | | LEELDRENLANR | 0,56 | 1 |
| AT2G20990 | | SYT1 | Synaptotagmin | | TLDGGEDGQPPDKYR | 0,51 (0,010) | 4 |
| AT1G61250 | | SC3 | Secretory carrier | | LSPLPPEPVGFDYGR | 0,49 (0,015) | 3 |
|  | |  |  | | TVDIPLDR | 0,50 (0,040) | 3 |
|  | |  |  | |  |  |  |
| ***Cell-wall related proteins*** | | | | | | | |
| AT2G04780 | | FLA7 | Fasciclin-like arabinogalactan-protein | | FTDVSGTVR | 0,53 (0,078) | 15 |
| AT2G45470 | | FLA8 | Fasciclin-like arabinogalactan-protein | | LADEINSR | 0,00 (0,000) | 11 |
|  | |  |  | | VGFGSAASGSK | 0,00 (0,000) | 9 |
| AT4G12420 | | SKU5 | Cu2^+^ binding, root tip growth | | ASGGFGSFVVNPR | 0,31 (0,010) | 6 |
|  | |  |  | | RPLTGPAK | 0,29 (0,010) | 3 |
| AT2G44790 | | UCC2 | Uclacyanin, blue copper protein | | VGDILEFK | 1,00 (0,000) | 9 |
|  | |  |  | | YGSSHTVDVVDK | 1,00 (0,000) | 34 |
| AT4G26690 | | SHV3 | Glycerophosphoryldiester phosphodiesterase | | FADAVVIQK | 1 | 1 |
| AT5G55480 | | SVL1 | Glycerophosphoryldiester phosphodiesterase | | SAAFDGNSYGISTVK | 0,41 | 1 |
|  | |  |  | | SVFPTSESFTTGQTK | 0,52 | 1 |
| AT1G66970 | | SVL2 | Glycerophosphoryldiester phosphodiesterase | | FANAVVINK | 0,00 (0,000) | 8 |
| AT3G04010 | |  | O-Glycosyl hydrolase, family 17 | | SVAPGNFER | 0,49 (0,004) | 5 |
|  | |  |  | |  |  |  |
| ***Signal transduction and stress responses*** | | | | | | | |
| AT3G08510 | | PLC2 | Phosphoinositide-specific phospholipase C | | AFPLHSR | 0,43 (0,027) | 16 |
|  | |  |  | | EDAQSIINSASSLLHR | 0,00 (0,000) | 2 |
|  | |  |  | | EFPSPNSLKR | 0,45 (0,014) | 2 |
|  | |  |  | | EVPSFIQR | 0,43 (0,044) | 10 |
|  | |  |  | | FLIDVQK | 0,44 | 1 |
|  | |  |  | | FTQHNLLR | 0,47 (0,024) | 14 |
|  | |  |  | | HTHFDQYSPPDFYTR | 0,47 (0,015) | 4 |
|  | |  |  | | INAPPQYK | 0,5 | 1 |
|  | |  |  | | RLSLSEEQLEK | 0,46 (0,014) | 2 |
|  | |  |  | | YTASEAPR | 0,47 | 1 |
| AT3G19820 | | DWF1 | Brassinosteroid biosynthesis | | GEEFDGSEAVR | 0,7 | 1 |
|  | |  |  | | GQIYPEPGFEYENR | 0,70 (0,006) | 3 |
| AT3G48890 | | MSBP2 | Progesterone binding protein | | GQIYDVSQSR | 0,40 (0,023) | 3 |
| AT5G06320 | | NHL3 | Similar to hairpin-induced (tobacco) | | DLNEDVNSQIYR | 0 | 1 |
| AT1G30360 | | ERD4 | Early responsive to dehydration | | AEAILAATNNRPTNK | 0,36 (0,017) | 10 |
|  | |  |  | | AEGIPSQSHAIR | 0,38 (0,011) | 8 |
|  | |  |  | | EFIDSYFR | 0,37 (0,01) | 4 |
|  | |  |  | | EIYPETFYR | 0,37 (0,008) | 7 |
|  | |  |  | | GLEPWEGTSLTR | 0,41 (0,01) | 3 |
|  | |  |  | | KSGNAPIYYPNR | 0,31 | 1 |
|  | |  |  | | QQTAAVVFFTTR | 0,39 (0,021) | 2 |
|  | |  |  | | SGNAPIYYPNR | 0,38 (0,011) | 10 |
|  | |  |  | | VYVPSYESYGR | 0,37 (0,013) | 4 |
| AT1G63500 | |  | Protein kinase | | ALYHDLNAYR | 0,50 (0,024) | 4 |
|  | |  |  | | HIPPSHALDLIR | 0,71 | 1 |
|  | |  |  | | VLFDDDSNPR | 0,54 (0,007) | 2 |
| AT3G51330 | |  | Aspartyl protease | | TTILFPR | 0,82 | 1 |
| AT4G04720 | | CPK21 | Ca2+-dependant protein kinase | | SIPINPVQTHVVPEHR | 0,60 (0,010) | 3 |
| AT5G53560 | | B5-A | Cytochrome b5 | | DATNDFEDVGHSDTAR | 0,51 (0,022) | 7 |
|  | |  |  | | YFIGEIDSSSVPATR | 0,52 (0,013) | 5 |
| AT2G37710 | | LRK1 | L-Lectin (LEC) RLK, response to salicylic acid | | TGHAFYTKPIR | 1 | 6 |
| AT3G02880 | |  | LRR III (5) RLK | | VSDYGLAPIISSTSAPNR | 0,63 | 1 |
|  | |  |  | | YQPEGNENIIR | 0,62 | 1 |
|  | |  |  | |  |  |  |
| ***Unknown*** | | | | | | | |
| AT1G58270 | | ZW9 | Unknown molecular functions | | YQISQETEAQR | 1,00 (0,000) | 2 |
| AT2G39530 | |  | Unknown molecular functions | | APPPPAPPSVTLR | 1,00 (0,000) | 13 |
| AT4G15610 | |  | Unknown molecular functions | | NIFLPGTPIR | 0,81 (0,017) | 12 |
| AT5G44550 | |  | Unknown molecular functions | | VGNTPIQATFTAK | 1,00 (0,000) | 7 |

^a^ Unique peptide sequences for which reliable ^14^N:^15^N spectra could be obtained. ^b^The distribution of a peptide between root and leaf plasma membrane (q-value of 0 means protein distribution only in leaf and q-value of 1 only in root). SD is the standard deviation for the q-value of the peptide based on all spectra with the ^14^N:^15^N peptide pair for that specific peptide. ^c^Number of MS spectra containing reliable data for ^14^N:^15^N peptide pair used to determine q-value. All spectra have fulfilled all criteria for that specific peptide/protein including correct position on SDS-gel, consistent elution profile on LC, correct molecular weight as well as isotopic pattern and finally a manual inspection of the spectra. *The q-value of Syntaxin (SYP 71) (3G09740) were identified with two unique peptides found in three different areas on the SDS gel, all areas with different q-values, thus no q-value for each peptides was determined.
